# Invasive plant species in the West Indies: geographical, ecological, and floristic insights

**DOI:** 10.1002/ece3.2984

**Published:** 2017-04-28

**Authors:** Julissa Rojas‐Sandoval, Raymond L. Tremblay, Pedro Acevedo‐Rodríguez, Hilda Díaz‐Soltero

**Affiliations:** ^1^ Department of BotanyNational Museum of Natural HistorySmithsonian InstitutionWashingtonDCUSA; ^2^ Center for Applied Tropical Ecology and ConservationUniversity of Puerto RicoSan JuanPRUSA; ^3^ United States Department of AgricultureWashingtonDCUSA

**Keywords:** anthropogenic disturbance, biogeography, biological invasions, Caribbean islands, invasiveness, taxonomy

## Abstract

The level of invasion (number or proportion of invasive species) in a given area depends on features of the invaded community, propagule pressure, and climate. In this study, we assess the invasive flora of nine islands in the West Indies to identify invasion patterns and evaluate whether invasive species diversity is related to geographical, ecological, and socioeconomic factors. We compiled a database of invasive plant species including information on their taxonomy, origin, pathways of introduction, habitats, and life history. This database was used to evaluate the similarity of invasive floras between islands and to identify invasion patterns at regional (West Indies) and local (island) scales. We found a total of 516 alien plant species that are invasive on at least one of the nine islands studied, with between 24 to 306 invasive species per island. The invasive flora on these islands includes a wide range of taxonomic groups, life forms, and habitats. We detected low similarity in invasive species diversity between islands, with most invasive species (>60%) occurring on a single island and 6% occurring on at least five islands. To assess the importance of different models in predicting patterns of invasive species diversity among islands, we used generalized linear models. Our analyses revealed that invasive species diversity was well predicted by a combination of island area and economic development (gross domestic product per capita and kilometers of paved roadways). Our results provide strong evidence for the roles of geographical, ecological, and socioeconomic factors in determining the distribution and spread of invasive species on these islands. Anthropogenic disturbance and economic development seem to be the major drivers facilitating the spread and predominance of invasive species over native species.

## 1 Introduction

Vascular plants are among the most common invasive organisms, as they are often introduced for agricultural, agroforestry, and ornamental purposes (D'Antonio, Jackson, Horvitz, & Hedberg, 2004; Daehler, 2003; Pyšek et al., 2012). The probability of introduction, establishment, and spread of invasive plant species (the introduction–naturalization–invasion continuum) is affected by geographical, ecological, and socioeconomic features of the invaded area (i.e., climate, resource availability, and demand for certain goods and services; Catford, Jansson, & Nilsson, 2009; Pyšek & Richardson, 2006; Reaser et al., 2007; Richardson & Pyšek, 2012), by external factors such as the initial population size, residence time, and propagule pressure (Catford, Vesk, White, & Wintle, 2011; Lockwood, Cassey, & Blackburn, 2005; Trueman et al., 2010), and by inherent species traits influencing its ability to survive, reproduce, disperse, and interact with other species, native or alien, already resident in the area (Pyšek & Richardson, 2006; Rojas‐Sandoval & Acevedo‐Rodríguez, 2015).

Islands provide exceptional systems for testing hypotheses on biological invasions (Denslow, Space, & Thomas, 2009; Kueffer et al., 2010; Simberloff, 2000). Island communities have been regarded as more vulnerable to biological invasions than continental ecosystems due to their relatively higher numbers and densities of alien species (D'Antonio & Dudley, 1995; Denslow, 2003; Pyšek & Richardson, 2006; Sax, Gaines, & Brown, 2002). Nevertheless, this apparent vulnerability of islands to biological invasion, which has been considered as one of the “classic” generalizations in invasion ecology, remains debatable. The high rates of invasibility and naturalization of alien plant species observed on temperate and tropical islands have been traditionally explained by inherent features of islands such as their geographical and historical isolation, high habitat diversity, disharmonic and unsaturated floras, low biotic resistance, and low pest pressures, which are factors that would provide limited barriers to the establishment and naturalization of alien plants (D'Antonio & Dudley, 1995; Denslow, 2003; Mack et al., 2000; Simberloff, 1995). However, these assumptions have been rarely supported by empirical data and other insular features such as small territories, reduced habitats, and small population sizes have been proposed as alternative explanations (Simberloff, 1995, 2000). Although the susceptibility to biological invasion does not necessarily coincide with the vulnerability to the deleterious effects caused by the spread of alien species, there is a consensus that islands are more susceptible to the effects of invasive species than continental ecosystems (D'Antonio & Dudley, 1995; Pyšek et al., 2012; Reaser et al., 2007). Insular biotas are considered to be fragile ecosystems characterized by high levels of endemism (Acevedo‐Rodríguez & Strong, 2008; Whittaker & Fernández‐Palacios, 2008). On these biotas, the introduction of alien species could lead to changes in community structure and the loss of native species, ecosystem functions, and services (Reaser et al., 2007; Rojas‐Sandoval, Meléndez‐Ackerman, & Anglés‐Alcázar, 2016; Spotswood, Meyer, & Bartolome, 2012). In many cases, these changes lead to taxonomic and functional simplification, a phenomenon known as biotic homogenization, which has been extensively investigated for oceanic islands (Castro et al., 2010; Castro & Jaksic, 2008; Stohlgren et al., 2011; Vergara, Pizarro, & Castro, 2011).

The West Indies, comprising the Bahamas, the Greater and Lesser Antilles, and the islands off the coast of northern South America (Netherlands Antilles and Trinidad and Tobago), is considered one of the biodiversity hotspots with high global priority for conservation due to its biological richness and high levels of endemism (>70% for seed plants; Myers, Mittermeier, Mittermeier, da Fonseca, & Kent, 2000; Mittermeier, Myers, Thomsen, da Fonseca, & Olivieri, 1998; Acevedo‐Rodríguez & Strong, 2012). However, in spite of such diversity, islands in the West Indies have been subjected to extreme levels of anthropogenic disturbance and massive degradation of their natural environments since the beginning of European colonization (Grove, 1996; Lugo, 2004; Maunder et al., 2008). Currently, the expansion of urban areas and tourism complexes coupled with high population densities and accelerated changes in socioeconomic conditions are generating unprecedented pressure over the natural resources on these islands (Dixon, Hamilton, Pagiola, & Segnestam, 2001).

In this study, we used data on invasive plant species collected on nine island groups to identify patterns of invasion across the West Indies. Although several studies and checklists of alien and invasive plants have been produced for some of these islands (Kairo, Ali, Cheesman, Haysom, & Murphy, 2003; Oviedo et al., 2012; Rojas‐Sandoval & Acevedo‐Rodríguez, 2015; Van der Burg, Freitas, Debrot, & Lotz, 2012), there have been no systematic studies comparing the patterns of invasion at the regional scale. Moreover, assessments of factors influencing invasibility and invasiveness are still limited for the West Indies (but see Ackerman et al., 2014; Ackerman, Tremblay, Rojas‐Sandoval, & Hernández‐Figueroa, 2017; Rojas‐Sandoval & Acevedo‐Rodríguez, 2015). Within this context, the main goals of this work were to: (1) describe the invasive flora of nine island groups across the West Indies, (2) evaluate the similarity of the invasive floras between islands, and (3) identify and evaluate geographical, ecological, and socioeconomic factors that best correlate with the invasive species diversity on these islands. The results derived from this study provide a general perspective of the patterns of distribution of invasive species within the Caribbean region and may serve as a base for future comparative studies on plant invasions and the design of management strategies to control and mitigate the impact of invasive plants on these islands.

## 2 Methods

### 2.1 Study site

We collected information on the presence of invasive plant species for the following nine island groups and/or political units: (1) Bahamas, (2) Cuba, (3) Dominican Republic, (4) Jamaica, (5) Puerto Rico, (6) Virgin Islands (including the British and the U.S. Virgin Islands), (7) St. Lucia, (8) St. Martin (including the Dutch and French parts of the island), and (9) Trinidad and Tobago (Table 1). These island groups (hereafter *islands*) are characterized by a wide variety of environmental, geographical, and socioeconomic attributes (Table 1). The largest island included in this study is Cuba (109,820 km^2^), and the smallest island is St Martin (88 km^2^). In the case of the British and the U.S. Virgin Islands, we decided to aggregate the information as a single unit because they share similar geographical and ecological histories and thus similar microclimates, habitats, and plant diversity (Acevedo‐Rodríguez & Strong, 2012).

**Table 1:** Geographical, ecological, and socioeconomic data for the nine island groups studied in the West Indies

Islands	Geographical		Ecological		Socioeconomic			
	Area (km^2^)	Highest elevation (m)	Forest cover (%)^a^	Native Species^b^	Population	Population density^e^	GDP (US$)^f^	Paved roadways (km)^g^
Bahamas	10,010	63	51.4	1,068^c^	324,597	32.4	25,100	1,620
Cuba	109,820	2,005	27.3	5,778^c^	11,031,433	100.5	10,200	29,820
Dominican Republic	48,320	3,175	40.8	2,896^c^	10,478,756	216.9	14,000	9,872
Jamaica	10,831	2,256	31.1	2,495^c^	2,950,210	272.4	8,600	16,148
Puerto Rico	8,870	1,338	63.2	2,108^c^	3,598,357	405.7	28,500	26,862
Virgin Islands	497	521	40.9	1,003^c^	137,028	275.7	39,200	1,860
St Lucia	606	950	77	1,079^c^	163,922	270.5	11,600	847
St Martin	88	424	50	409^c^	71,443	808.2	43,050	53
Trinidad&Tobago	5,158	940	44	2,086^d^	1,222,363	238.4	32,200	4,252

^a^Percentage of total land area covered by forests. Forest area is land spanning more than 0.5 hectare with trees higher than five meters and a canopy cover of more than 10% to include windbreaks, shelterbelts, and corridors of trees greater than 0.5 hectare and at least 20 m wide. ^b^Including only spermatophyte species, ^c^Acevedo‐Rodríguez & Strong, 2012 and ^d^Trinidad and Tobago Biodiversity (http://www.biodiversity.gov.tt/home/). ^e^People per km^2^. ^f^Gross domestic product (GDP) per capita on a purchasing power parity in US dollars. ^g^Total length of the road network with paved portions.

### 2.2 Data collection

Our database of invasive species was compiled from published literature including research articles, dissertations, and reports from local governments and conservation organizations (see Appendix S1 in Supporting Information for bibliographic references). We only included alien spermatophyte species classified as “invasive” on the original sources consulted. These sources followed standardized criteria (e.g., Beck et al., 2008; Richardson et al., 2000) to classify species as invasive: (1) species are non‐native, (2) they have established in new habitats without direct human assistance after their introduction, and (3) they are spreading over considerable areas causing negative (economic or environmental) impacts. We verified the invasive status and filtered the data by examining additional sources (specialist revisions, scientific publications, and herbarium collections). Species occurring exclusively in human‐made habitats such as gardens and parks were excluded from the analyses. Similarly, species with unreliable records or uncertain origin (i.e., listed as both native and alien) were also excluded. The species entries were supplemented with data on their taxonomy, native distribution, life form (aquatic herb, grass, herb, shrub, succulent, tree, vine), life history (annual, biennial, perennial), type of introduction (intentional, unintentional, escaped), and planting purposes/uses (ornamental, agriculture/food crops, forage/fodder, agroforestry, soil improver, timber tree) obtained from a list of data sources included in Appendix S2. Habitat categories were used to classify species occurrence in: agricultural habitats (traditional agriculture lands), ruderal (human‐made habitats, excluding agriculture lands), seminatural (low to medium disturbed areas, including secondary forest), and mature forest (very low disturbed areas) (Lloret et al., 2005; Rojas‐Sandoval & Acevedo‐Rodríguez, 2015). Note that some species can occur in more than one habitat or can be assigned to several planting purposes/uses categories. Similarly, if the native distribution of a species covers more than one continent, it is assigned to each of them.

### 2.3 Characterization of islands

Geographical, socioeconomic, and ecological data that were suspected drivers of invasive species diversity were assessed for each island. The complete list of variables assessed is included in Table 1. These data were obtained in August 2016 from The World Factbook (https://www.cia.gov/library/publications/the-world-factbook/) and from the UNEP Island Directory (http://islands.unep.ch/). Data for islands with political units corresponding to different countries (e.g., Dutch and French parts of St Martin and the British and U.S. Virgin Islands) were combined and reported as absolute or mean values depending on the variable type (Table 1).

### 2.4 Data analysis

To evaluate patterns of similarity on invasive species richness among islands, we calculated Sørensen coefficients as *S*_ø_ *= *(*2C/A + B*)*100, where *A* and *B* are the number of invasive species found on island A and B, respectively, and *C* is the number of invasive species common to both islands (McGarigal, 2000). The *S*_ø_ coefficient varies between 0 (completely different) and 100 (identical). Additionally, to test if there was a correlation between similarity coefficients and the geographical distance among islands, we used a Mantel test comparing the matrix of *S*_ø_ values for all island pairs and a matrix of corresponding geographical distances. The statistical significance of the resultant correlation was determined by a Monte Carlo simulation with 10,000 permutations (Legendre & Vaudor, 1991). Generalized linear models (GLM) were used to assess the relationships between invasive species diversity and geographical, socioeconomic, and environmental factors among islands. In these analyses, the factors evaluated as explanatory variables were the following: island area, elevation, forest cover, population density, gross domestic product (GDP) per capita, and kilometers of paved roadways (Table 1), while the number of invasive species per island and the ratio of invasive to native species were used as dependent variables. When the number of invasive species per island was evaluated as the dependent variable, we used GLM with a negative binomial model, but when the ratio of invasive to native species was the depended variable, we used GLM with a beta regression model. The beta regression method is an extension of the GLM and it is especially meant for modeling rates and proportions, as these data are non‐normally distributed, while the negative binomial method is more appropriate for count data with a variance that is greater than the mean (McCullagh & Nelder, 1989). We started by creating a “full model” including all variables together, and then new models were created by simplifications of the full model to evaluate explanatory variables. As a result, a total of 64 models including all possible explanatory variable combinations were created for each dependent variable. All models evaluated are included in Appendices S3 and S4. Model selection was performed using the corrected Akaike's information criterion (AICc), a modification of the AIC that include a bias correction term for small sample size (Burnham & Anderson, 2002). In general, the model with the smallest AICc is considered the best model and AICc differences between 0 and 2 suggest weak differences among the models while differences larger than 4 are considered to be large (Arnold, 2010). All analyses were carried out in the R statistical program (R Development Core Team, 2009), using the *glm.nb* function in MASS package and *betareg* function in the betareg package in conjunction with the *dredge* function in the MuMin package for comparing all models.

## 3 Results

**Figure 1:**
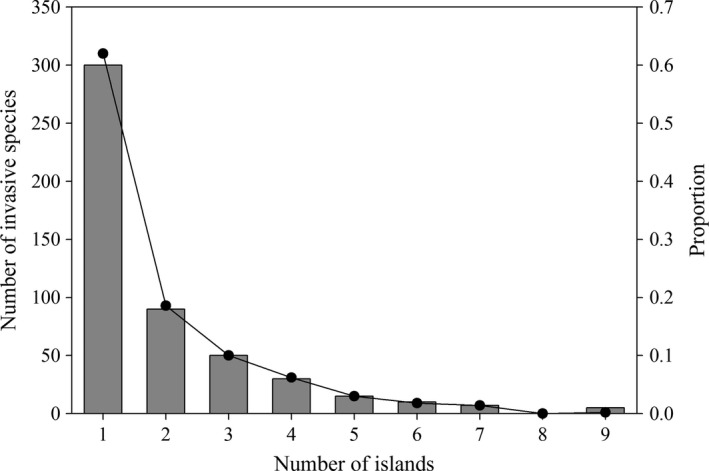
Frequency distribution of occurrences of invasive plant species across nine islands in the West Indies. The dot track represents the proportion relative to the total number of invasive plant species for all islands pooled (total = 516 species)

The final database comprises a total of 516 invasive plant species representing 348 genera and 96 families and contributing to 4% of the total flora of the West Indies (Table S1). This database comprises a heterogeneous group of plants in terms of their taxonomical and ecological attributes (Table S2). The families with the largest number of invasive species were Fabaceae (88 species), Poaceae (77 species), and Asteraceae (33 species), which accounted for 38% of all invasive species. A total of 34 families were represented by a single species and 78 families were represented by five species or less. The genera with the highest number of invasive species were *Ipomoea* (10 species), *Paspalum* (7 species), and *Eragrostis* (6 species), while 252 genera (72%) were represented by a single invasive species (Table S2). The frequency distribution of invasive plant species across islands is right‐skewed (Figure 1), with more than 60% (310 species) of all species only occurring on one island and 6% (32 species) occurring on five islands or more. The most widely distributed invasive species across the West Indies is *Leucaena leucocephala* (Fabaceae) which occurs on all nine islands, followed by *Casuarina equisetifolia* (Casuarinaceae)*, Eichhornia crasssipes* (Pontederiaceae)*, Megathyrsus maximus* (Poaceae)*, Melaleuca quinquenervia* (Myrtaceae)*, Ricinus communis* (Euphorbiaceae)*, Spathodea campanulata* (Bignoniaceae), and *Terminalia catappa* (Combretaceae), which occur on seven of nine islands (Table S3).

**Figure 2:**
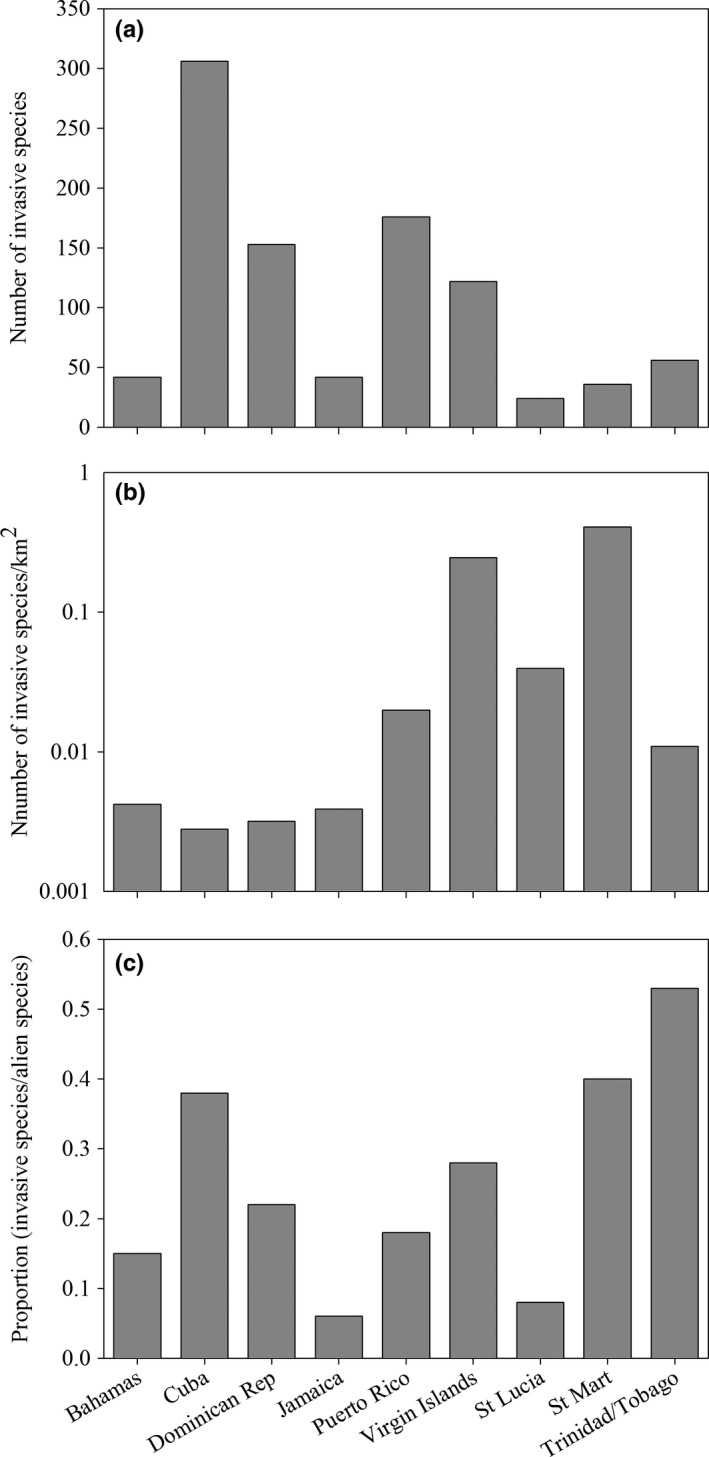
Distribution of (a) the number of invasive plant species, (b) the density of invasive plant species (number of invasive plants per km^2^), and (c) the proportion of alien species becoming invasive on each island. Note that the *y*‐axis in panel b is expressed in logarithmic scale

At the island scale, invasive species showed considerable variation in diversity (Figure 2; Table S1). The island with the lowest number of invasive species was St Lucia (24 species), while Cuba with 306 species was the island with the highest number of invasive species (Figure 2a). However, when evaluating invasive species diversity relative to island area, we found that St Martin has the highest density of invasive species (0.407 species per km^2^) followed by the Virgin Islands (0.245 species per km^2^), whereas Dominican Republic (0.003 species per km^2^) and Cuba (0.002 species per km^2^) showed the lowest density values (Figure 2b). When comparing the number of invasive species relative to the total number of alien plant species reported for each island (Figure 2c; Table S1), Trinidad and Tobago was found to have the highest proportion of alien plant species that have become invasive (54%), followed by St Martin (40%) and Cuba (38%). In contrast, Jamaica (6%) and St Lucia (8%) are the islands with the lowest proportion of alien plant species becoming invasive. At the regional scale, the 516 invasive species included in our database represent 27% of all alien plant species recorded for the West Indies (ca. 1,879 alien plant species, Table S1).

### 3.1 Similarity of invasive species among islands

The nine islands analyzed show different patterns of similarities relative to their invasive species richness, with Sørensen coefficients varying from 81.9 to 2.5 (average = 21, *SD *= 12; Table 2). Puerto Rico and the Virgin Islands are the most similar islands, sharing 122 invasive species, while St Lucia and Trinidad and Tobago are the most dissimilar, sharing only a single species (Table 2). The Mantel test showed no significant correlation between Sorensen's index and geographical distance among islands (*r *=* *−.34; *p *=* *.97). In general, the low Sørensen coefficients (<35.3) detected for most island pairs (except for Puerto Rico and the Virgin Islands) suggest that invasive floras are highly dissimilar among islands.

**Table 2:** Matrix of Sørensen similarity coefficients for invasive plant species on nine islands in the West Indies. Entries in the upper half are Sørensen similarity coefficients while entries in the lower half correspond to the number of invasive plant species shared between each pair of islands. Highest and lowest values are underlined

Islands	Bahamas	Cuba	Dominican Republic	Jamaica	Puerto Rico	Virgin Islands	St Lucia	St Martin	Trinidad & Tobago
Bahamas	–	16.1	16.4	26.2	21.1	25.6	18.2	23.1	12.1
Cuba	28	–	28.8	11.5	35.3	29.9	9.1	17.0	11.6
Dominican Rep	16	66	–	19.5	33.4	33.5	10.2	21.2	18.1
Jamaica	11	20	19	–	16.5	15.9	12.1	10.3	18.2
Puerto Rico	23	85	55	18	–	81.9	19.0	22.6	22.3
Virgin Islands	21	64	46	13	122	–	19.2	29.1	23.5
St Lucia	9	15	9	4	19	14	–	6.7	2.5
St Martin	9	29	20	4	24	23	2	–	19.4
Trinidad & Tobago	6	21	19	9	26	21	1	9	–

### 3.2 Life forms and habitats

Invasive plant species in the West Indies show diverse life forms, with a prevalence of herbs (162 species, 31%) followed by trees (102 species, 20%), shrubs (80 species, 16%), grasses (77 species, 15%), vines (67 species, 13%), succulents (15 species, 3%), and aquatic herbs (13 species, 2.5%). The majority of the invasive species (77%) are perennials, while the remaining 23% are considered annuals (Table 3). At the island scale, herbs are again the dominant life form on five of the nine islands analyzed (Cuba, Dominican Republic, Puerto Rico, Virgin Islands, and St Lucia), followed by trees that are the dominant life form on three islands (Bahamas, Jamaica, and Trinidad and Tobago) and grasses that are the dominant life form on St Martin (Table 3). Across the nine islands, invasive species occur in all four habitats considered in this study: ruderal (506 species, 98%), seminatural (314 species, 61%), agricultural (138 species, 35%), and mature forests (27 species, 5%), with species often found in more than one habitat. These results showed that for the West Indies invasive plant species are considerably more frequent in ruderal and seminatural habitats regardless of whether they also occur on other habitats.

**Table 3:** Number of invasive plant species of different life forms and life histories for each island and for all islands pooled

Island	Life form							Life history	
	Aquatic herb	Grass	Herb	Shrub	Succulent	Tree	Vine	Annual	Perennial
Bahamas	1	3	4	6	1	15	12	2	40
Cuba	11	56	85	48	14	43	49	68	238
Dominican Republic	4	18	52	26	4	37	12	41	112
Jamaica	2	7	7	7		18	1	4	38
Puerto Rico	2	35	54	19	5	33	28	43	133
Virgin Islands	1	21	34	13	4	27	22	32	90
St Lucia	3	1	7	5	1	3	4		24
St Martin		9	5	5	4	7	6	8	28
Trinidad & Tobago		9	12	6		27	2	15	41
All islands	13	77	162	80	15	102	67	121	395

### 3.3 Geographical distribution and pathways of introduction

**Figure 3:**
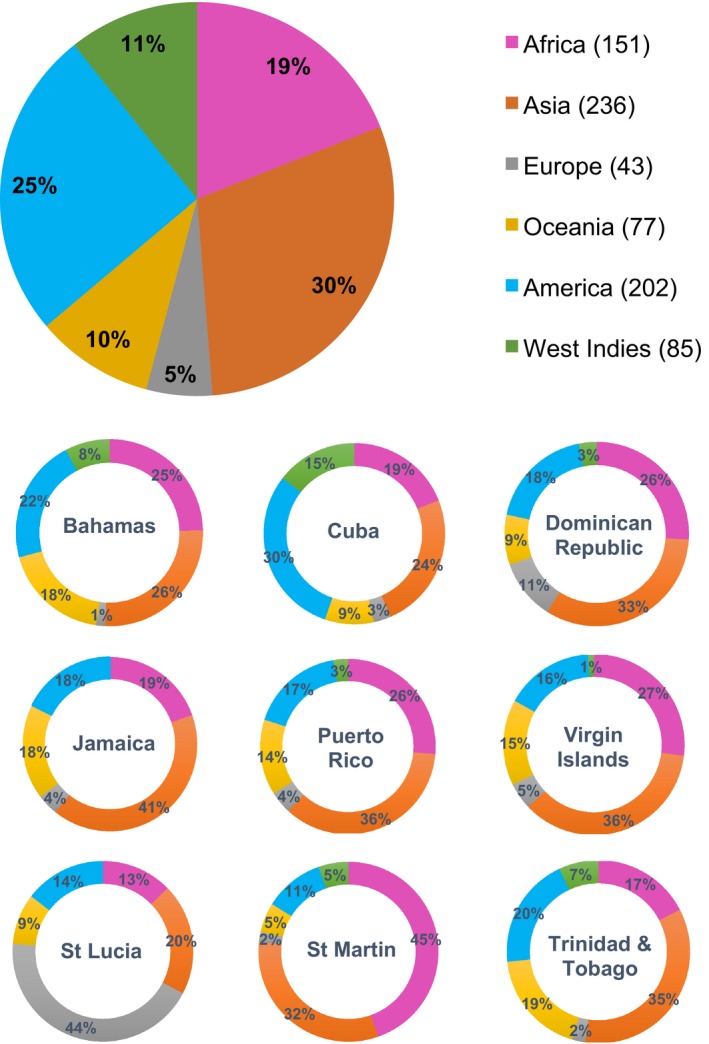
Donor regions of the invasive plant species in the West Indies. The percentages in the charts represent the contribution of each region to the total number of invasive species on each island and for all islands pooled. The number in parentheses denotes the number of species native to each region. Species with native distribution in more than one continent were assigned to each of the continents

Invasive plant species in the West Indies can be sourced to all continents (Figure 3), with the largest fraction coming from Asia (30%), followed by species coming from continental America (25%) and Africa (19%). Asiatic species are largely represented in the invasive flora of the Bahamas, Cuba, Dominican Republic, Jamaica, Puerto Rico, Virgin Islands, and Trinidad and Tobago. Among the nine islands studied, Cuba has the highest percentage of invasive species coming from continental America (30%). The islands of St Martin and St Lucia, on the other hand, have the highest percentage of invasive species derived from Africa (45%) and Europe (44%), respectively (Figure 3). When considering the type of introduction, we found that 75% of all invasive plants are species that have escaped from cultivation, 18% are the result of unintentional introductions (e.g., contaminants), and 7% were deliberately introduced into natural ecosystems (e.g., plants for erosion control). The most common planting purpose/use category was ornamental (51%) followed by species introduced for forage and fodder production (12%), agriculture/food crops (11%), agroforestry (6%), soil improvement (5%), and timber production (2%).

### 3.4 Factors influencing invasive species diversity

**Figure 4:**
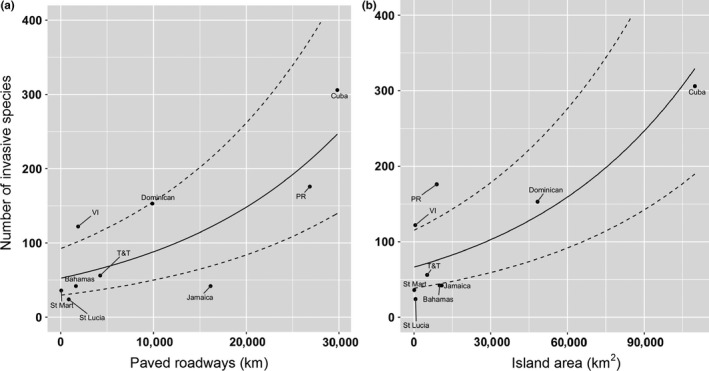
Number of invasive plant species as a function of (a) kilometers of paved roadways and (b) island area. These are the visualizations of the best‐fit models predicted by the GLM with negative binomial models. The median is indicated by the solid line and 95% percentile confidence intervals are indicated by dashed lines. T&T, Trinidad and Tobago; VI, Virgin Islands; Dominican, Dominican Republic; PR, Puerto Rico; St Mart, St Martin

Our GLM analyses for the number of invasive species as the dependent variable showed that the model including all factors explained 36% of the variation (Table 4; Fig. S1). However, the AICc analyses for determining the simplest best‐fit model showed that from all 64 models created, only two equivalent models (∆AICc < 2.0; Appendix S3) were good predictors of the number of invasive species per island. The first model included only the kilometers of paved roadways (AICc = 104.20; AIC_weight_ = 0.42; Figure 4a), while the second model included only the island area (AICc = 105.52; AIC_weight_ 0.22; Figure 4b). The AICc statistics suggest that both models are robust (Table 4). The third best model included only elevation (AICc = 108.96; AIC_weight_ = 0.04), but it was less well supported than the two best‐fit models and the difference in AICc was larger than 4 (Table 4).

**Table 4:** Coefficients estimated from the generalized linear models. Coefficients for the number of invasive species as the dependent variable were estimated with negative binomial models. Coefficients for the ratio of invasive to native species as the dependent variable were estimated with beta regression models. Model results are arranged by AICc values. MA: model including all factors. M1, M2, M3: the three best‐fit models based on the AICc. *Coefficient values are significant at *p *<* *.05

	Geographical		Ecological	Socioeconomic			AICc	Δ AICc
	Area (km^2^)	Elevation (m)	Forest cover (%)	Population density	GDP (US$)	Paved roadways (km)		
Models for number of invasive species								
M1						5.19e‐5*	104.20	–
M2	1.46e‐5*						105.52	1.32
M3		4.64e‐4*					108.96	4.76
MA All factors	1.18e‐5*	5.45e‐4*	1.10e‐2	–2.01e‐3*	7.51e‐5*	3.89e‐5*	213.58	109.38
Models for ratio invasive/native								
M1					3.38e‐5*		−32.56	–
M2				1.15e‐3			−28.80	3.76
M3	9.42e‐6				4.77e‐5		−28.35	4.21
MA All factors	1.04e‐5	2.43e‐4	1.33e‐2	−3.52e‐4	6.96e‐5*	9.60e‐6	84.40	116.96

**Figure 5:**
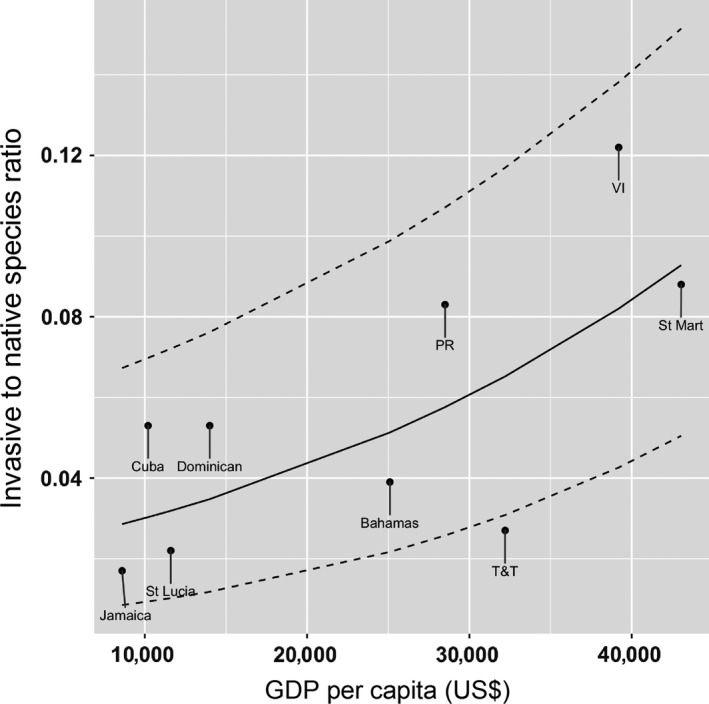
Invasive to native species ratio as a function of the gross domestic product (GDP) per capita in US dollars. This is the visualization of the best‐fit model predicted by the GLM with beta regression models. The median is indicated by the solid line, and 95% percentile confidence intervals are indicated by dashed lines. T&T, Trinidad and Tobago; VI, Virgin Islands; Dominican, Dominican Republic; PR, Puerto Rico; St Mart, St Martin

Using the ratio of invasive to native species as the dependent variable, the GLM analyses showed that the model including all factors explained 75% of the variation (Table 4; Fig. S1). Nevertheless, from all 64 models generated (Appendix S4), the simplest best‐fit (most parsimonious) model (AICc = −32.56; AIC_weight_ = 0.39; Figure 5) included only one variable: the GDP. The next best models included human population density (AICc = −28.80; AIC_weight_ 0.06) and a combination of island area and GDP (AICc = −28.35; AIC_weight_ = 0.05) but both were less well supported than the best‐fit model (Table 4). Overall, these results suggest that the number of invasive species increases consistently with island size and that paved roadways (an index of economic development) are facilitating the entrance and movement of invasive species on these islands. On the other hand, the ratio of invasive to native species increases with economic development and human population density, suggesting that human activities are favoring the establishment of invaders over native species.

## 4 Discussion

We have identified a total of 516 alien plant species that are invasive on at least one of the nine islands studied. The incidence of invasive plant species in the West Indies seems to be high when compared to the numbers reported for other island groups. For instance, Kueffer et al. (2010) studied 30 island groups worldwide and reported 383 invasive plants species, and Weber (2003) estimated 448 invasive plants globally for islands and continents combined. However, these studies only included alien plant species invading natural terrestrial habitats while our study included natural terrestrial and aquatic habitats, but also ruderal and semi‐natural habitats. Indeed, for the nine islands included in our study, the majority of invasive species occur (but not exclusively) on ruderal and disturbed habitats. Our results show that the proportion of alien species that have become invasive, at both regional and local scales, is also higher than the corresponding value reported for other islands: This proportion ranges from 6% to 54% on selected islands across the West Indies (this study), while the values reported for other islands are generally lower and vary from 3% in the Juan Fernandez Archipelago (Castro & Jaksic, 2008) and 5% on Hawaii Islands, to 11% in Fiji (Meyer, 2014) and 12% on Galapagos Islands (Trueman, Atkinson, Guézou, & Wurm, 2010).

The high number of invasive species as well as the high proportion of alien species that have become invasive in the West Indies is likely a combination of (1) the historic role of these islands as a hub for the Europe–America trading routes and (2) the early and widespread degradation of their natural habitats. For more than three centuries (1500s–1890s), European colonizers used the Caribbean islands as ports of call for trading routes between the Americas and the “Old World,” facilitating intentional and accidental introductions of numerous alien species coming from Asia, Africa, Europe, and continental America (Oviedo et al., 2012; Rojas‐Sandoval & Acevedo‐Rodríguez, 2015). Additionally, Caribbean islands have been subjected to high levels of anthropogenic disturbance first due to agriculture expansion and exploitation of their natural resources (e.g., logging), and more recently due to urban and tourism development (Acevedo‐Rodríguez & Strong, 2008; Dixon et al., 2001). Several studies have demonstrated that the level of disturbance (i.e., habitat quality) plays a crucial role in determining the level of invasion in invaded communities and that alien species are more likely to establish in disturbed habitats than in pristine habitats (Catford et al., 2011; Lockwood et al., 2005; Rojas‐Sandoval & Acevedo‐Rodríguez, 2015). In the case of the West Indies, islands are not only exposed to high levels of anthropogenic disturbances but also recurrently impacted by large‐scale natural disturbances such as hurricanes and tropical storms, which may drive changes in the structure, composition, and functioning of plant communities (Bellingham, Tanner, & Healey, 2005; Lugo, 2008; Walker, Lodge, & Waide, 1991). Consequently, it is plausible that the high disturbance rates prevailing on these islands are facilitating the successful establishment of invasive species populations (Lockwood et al., 2005; Richardson & Pyšek, 2012).

Our combined results indicate that the invasive floras of the West Indies are ecologically and taxonomically very diverse, with low similarity of invasive species richness observed among islands. Fabaceae and Poaceae are the families with the highest numbers of invasive species, as expected given that they are among the largest plant families at both global and local scales (Acevedo‐Rodríguez & Strong, 2012; Daehler, 1998; Pyšek, 1998) and they are the two most diverse families in the alien and invasive floras of other insular systems (Guézou et al., 2010; Kueffer et al., 2010; Silva & Smith, 2004). On Caribbean Islands, most Fabaceae and Poaceae invaders are species that have escaped from active and abandoned pastures and agroforestry systems (introduced primarily as nitrogen fixers, soil improvers, forages, and fodders) and ornamentals escaped from cultivation (Oviedo et al., 2012; Rojas‐Sandoval & Acevedo‐Rodríguez, 2015; Van der Burg et al., 2012). In fact, our results showed that most invasive plant species in the West Indies (75%) have escaped from cultivation. We also found that more than 50% of plant invaders were introduced as ornamentals on these islands. Ornamental trade has been recognized as the main pathway for plant invasions worldwide (Dehnen‐Schmutz, Touza, Perrings, & Williamson, 2007; Hulme, 2011) and has been shown to be a crucial factor explaining the establishment, naturalization, and subsequent invasion of alien plant species on Caribbean islands (Rojas‐Sandoval & Acevedo‐Rodríguez, 2015). The recurrent introduction of certain species associated with ornamental trade, directly linked to economic development (see below), may increase propagule pressure and the establishment probability of invasive species (Lockwood, Cassey, & Blackburn, 2009; Mack & Lonsdale, 2001; Pemberton & Liu, 2009; Reichard & White, 2001).

On islands, human activities are known drivers for the spread and establishment of invasive species (Kueffer et al., 2010; Reaser et al., 2007; Richardson & Pyšek, 2012). Our statistical models based on GLM show that invasive species diversity is well predicted by a combination of island area and economic development (measured here as GDP per capita and kilometers of paved roadways). For the West Indies, plant invasions appear to be more frequent on large islands with substantial economic activity. These patterns are in general agreement with other studies showing that measures of economic development including GDP, presence of airports, and trade intensity are good predictors of invasive species diversity on islands (Denslow et al., 2009; Kueffer et al., 2010). Indeed, economic development is often correlated with the magnitude of alien introductions as well as with the intensity of anthropogenic disturbance of natural areas (Kueffer et al., 2010; Pyšek & Richardson, 2006; Reaser et al., 2007). On islands with higher economic activity, the magnitude of introduction and planting (propagule pressure) of particular species, especially ornamentals and commodity plants, are expected to be higher than in less developed islands. Our results suggest that this link between propagule pressure and economic development may be crucial to predict the future trajectories of plant invasions in the West Indies. Critically, the high rates of invasibility on Caribbean islands come at the cost of negative impacts on native ecosystems and the threat of extinction of endemic species (Rojas‐Sandoval & Meléndez‐Ackerman, 2012; Rojas‐Sandoval, Meléndez‐Ackerman, & Fernández, 2012; Rojas‐Sandoval et al., 2016).

The distribution and abundance of invasive species can also be strongly influenced by vectors that facilitate their dispersal (Mortensen et al., 2009; Von der Lippe & Kowarik, 2012). Besides being an indirect measure of economic development, the presence of paved roadways can play a crucial role by serving as corridors for the spread of invasive species as well as by providing particularly propitious habitats (open and disturbed) for their establishment (Pauchard & Alaback, 2004; Sharma & Raghubanshi, 2009; Jolly et al., 2011; Vakhlamova, Rusterholz, Kanibolotskaya, & Baur, 2016). We found that the total length of paved roadways is indeed a good predictor of invasive species diversity for islands in the West Indies. Previous studies have also shown that paved roadways strongly favor the spread and establishment of opportunistic invasive plant species (Mortensen et al., 2009; Jolly et al., 2011; Meunier & Lavoie, 2012). From roadside areas, invasive species can also colonize adjacent undisturbed habitats (Pauchard & Alaback, 2004; Sharma & Raghubanshi, 2009; Von der Lippe & Kowarik, 2012). Vehicles are an important vector for plant dispersal, and road networks associated with higher traffic densities result in high propagule pressure of alien species (Ansong & Pickering, 2013; Von der Lippe & Kowarik, 2008). Overall, our models highlight the vulnerability of islands with high economic activity and large paved road networks to the introduction and spread of invasive plant species. This represents an underestimated threat to the already highly disturbed habitats on Caribbean islands.

## 5 Conclusions

Invasive species richness among islands in the West Indies is well predicted by a combination of island area and economic development including GDP per capita and kilometers of paved roadways. Low similarities in invasive species diversity between islands were detected, with most invasive species (>60%) occurring on a single island and only 6% occurring on at least five islands. Evidence suggests that species behaving as invasive in one area have higher probability to become invasive in other areas (Richardson & Pyšek, 2012). The dataset of invasive species presented here could thus be used as a blacklist of unwanted species to prevent their spread across the region and to avoid new introductions of likely invasive species. Overall, our data emphasize the need to limit deliberate introduction of alien species (particularly ornamentals) and reduce anthropogenic disturbance on these fragile ecosystems. Among the 36 plant species listed as the “world's worst invasive alien species” (Lowe, Browne, Boudjelas, & De Poorter, 2000), 19 species appeared in our dataset. Considering the extraordinary species richness and extent of endemism of the West Indies floras, the potential negative impact of these invasive species is very high (e.g., Rojas‐Sandoval et al., 2016). Future studies should consider complete floristic inventories of alien species, including casual and naturalized species that could eventually become invasive. These inventories are particularly relevant because they allow comparative regional analyses crucial for understanding invasion patterns. From a management perspective, there is an urgent need to implement more effective prevention policies at all scales and enforce more stringent national and regional legislations.

## Author contributions

JRS conceived and designed the study, compiled the database and prepared the data for analyses; JRS and RLT analyzed the data and created the figures. JRS wrote the manuscript including comments from all authors.
