# Supplementary material for: Invasive plant species in the West Indies: geographical, ecological, and floristic insights
Source: Ecol Evol. 2017 Apr 28;7(13):4522–33. doi: 10.1002/ece3.2984 (PMC5496547; doi:10.1002/ece3.2984)
Supplement: Supplementary file 1 [file ECE3-7-4522-s001.pdf]

Supporting Information

Invasive plant species in the West Indies: geographical, ecological and floristic insights

Authors: Julissa Rojas-Sandoval, Raymond L. Tremblay, Pedro Acevedo-Rodríguez & Hilda Díaz-Soltero.

Corresponding author: Julissa Rojas-Sandoval (julirs07@gmail.com)

Appendix S1. List of data sources used to compile the list of invasive plant species for nine islands in the West Indies.

Bahamas	BEST Commission (2003) The National Invasive Species Strategy for The Bahamas. BEST, Nassau, The Bahamas.<br><br>Smith, R.L. (2010) Invasive Alien Plant Species of the Bahamas and Biodiversity Management. Thesis. Institute of Environmental Sciences, Miami University, Oxford, Ohio.
Cuba	Oviedo, R., Herrera, P., Caluff, M.G., Regalado, L., Ventosa, I., Plasencia, J.M., Baró, I., González, P.A., Pérez, J., Hechavarría, L. & González-Oliva, L. (2012). Lista nacional de especies de plantas invasoras y potencialmente invasoras en la República de Cuba-2011. *Bissea*, 6, 22-96.
Dominican Republic	Mir, C. (2012). Estrategia Nacional de Especies Exóticas Invasoras Realizado en el marco del Proyecto "Mitigando las amenazas de las especies exóticas invasoras en el Caribe Insular". Ministerio de Medio Ambiente y Recursos Naturales Santo Domingo, República Dominicana.
Jamaica	Townsend, S. & Newell, D. (2006). Technical Progress Report. I3N Database in Jamaica. Natural History Division, Institute of Jamaica Kingston, Jamaica.
Puerto Rico and Virgin Islands	Rojas-Sandoval, J & Acevedo-Rodríguez, P. (2015). Naturalization and invasion of alien plants in Puerto Rico and the Virgin Islands. *Biological Invasions*, 17, 149-163.
St Lucia	Graveson, R. (2009). The Classification of the Vegetation of Saint Lucia. Technical Report No. 3 to the National Forest Demarcation and Bio-Physical Resource Inventory Project, FCG International Ltd, Helsinki, Finland.<br><br>Graveson, R. (2012). The Plants of Saint Lucia (in the Lesser Antilles of the Caribbean). www.saintlucianplants.com Accessed: 06/17/2016
St Martin	MacRae, D.R. & Nisbeth, B.M. (2008). St. Maarten Proposed Land Parks Management Plan 2008. Coastal Zone Management (UK), Nature Foundation St. Maarten.<br><br>Smith, S.R.. Van der Burg, W.J., Debrot, A.O., van Buurt, G. & Freitas, J. (2014). Key elements towards a joint invasive alien species strategy for the Dutch Caribbean. PRI report 550. Wageningen, The Netherlands.

	Van der Burg, W.J., Freitas, J., Debrot, A.O. & Lotz, L.A.P. (2012). Naturalised and invasive alien plant species in the Caribbean Netherlands: status, distribution, threats, priorities and recommendations. Plant Research International. PRI report 437. Wageningen, The Netherlands.
Trinidad/Tobago	Hosein, F. (2011). National Invasive Alien Species Strategy for Trinidad and Tobago. UNEP –GEF Project: Mitigating the Threat of Invasive Alien species in the Insular Caribbean. Trinidad and Tobago.<br><br>Trinidad and Tobago Biodiversity. (2016). Lists of Invasive species in Trinidad and Tobago. http://www.biodiversity.gov.tt/home/trinidad-a-tobago-biodiversity/invasive-alien-species.html Accessed: 06/17/2016
West Indies	CABI Invasive Species Compendium, Wallingford, UK: CAB international (http://www.cabi.org/isc/)<br><br>UICN-Global Invasive Species Database (http://www.iucngisd.org/gisd/)<br><br>Kairo, M., Ali, B., Cheesman, O., Haysom, K. & Murphy, S. (2003). Invasive Species Threats in the Caribbean Region – Report to The Nature Conservancy. CAB International, Curepe, Trinidad and Tobago & Egham, UK.

Appendix S2. List of data sources used to supplement the list of invasive plant species with information on taxonomy, life-history, habitat strategy, uses and pathways of introduction.

1	Acevedo-Rodriguez, P. (2005). Vines and climbing plants of Puerto Rico and the Virgin Islands. *Contribution of the United States National Herbarium*, **51**, 1–483.
2	Acevedo-Rodriguez, P. & Strong, M.T. (2005). Monocotyledons and gymnosperms of Puerto Rico and the Virgin Islands *Contribution of the United States National Herbarium*, **52**, 1–415.
3	Adams, C.D. (1972). *Flowering Plants of Jamaica*. University of West Indies, Mona, Jamaica.
4	Correll, D.S. & Correll, H.B. (1982). *Flora of the Bahamas Archipelago*. J. Cramer, FL-9490 Vaduz, Germany.
5	Howard, R.A. (1974-1989). *Flora of the Lesser Antilles*. Arnold Arboretum of Harvard University, Jamaica Plain, Massachusetts.
6	Liogier, A.H. (1985-1997). *Descriptive Flora of Puerto Rico and adjacent islands*. Spermatophyta 1-5. Editorial de la Universidad de Puerto Rico, Puerto Rico.
7	USDA-Plants database (http://plants.usda.gov)
8	USDA-GRIN database (http://www.ars-grin.gov/)

Table S1. Floristic estimates, ratios of invasion, and total contribution of invaders to the flora of nine islands in the West Indies.

	Total number of species[1]	*Native species[1]*	*Alien species[1]*	*Invasive species[2]*	*Invasive/alien species*	*Invasive/total number of species*
Islands						
Bahamas	1,337	1,068	269	42	0.156134	0.031414
Cuba	6,567	5,778	789	306	0.387833	0.046597
Dominican Rep	3,575	2,896	679	153	0.225331	0.042797
Jamaica	3,175	2,495	680	42	0.061765	0.013228
Puerto Rico	3,082	2,108	974	176	0.180698	0.057106
St Lucia	1,373	1,079	294	24	0.081633	0.01748
St Martin	493	409	89	36	0.404494	0.073022
Trinidad/Tobago	2,190	2,086	104	56	0.538462	0.025571
Virgin Islands	1,431	1,003	428	122	0.285047	0.085255
West Indies	12,280	10,401	1,879	516	0.274614	0.04202

[1]Acevedo-Rodriguez, P. & Strong, M.T. (2012). Catalogue of seed plants of the West Indies. Smithsonian Contributions to Botany, No. 98. Smithsonian Institution Scholarly Press, Washington DC. [2]Lists of invasive plants species created for this study.

Table S2. Summary of taxonomic information for the 516 invasive plant species recorded on nine islands in the West Indies. Data are grouped by island and for all islands pooled. The three largest families and genera with the most number of species are included. Number in parentheses denotes the number of invasive plants species.

Island	*Summary*	*Family*	*Genus*
Bahamas	26 families	Fabaceae (7)	*Casuarina* (2)
	38 genera	Myrtaceae (3)	*Dioscorea* (2)
	42 species	Poaceae (3)	*Ipomoea* (2)
Cuba	77 families	Poaceae (56)	*Ipomoea* (8)
	220 genera	Fabaceae (39)	*Paspalum* (5)
	306 species	Asteraceae (18)	*Eragrostis* (4)
Dominican Republic	51 families	Fabaceae (34)	*Acacia* (3)
	132 genera	Asteraceae (18)	*Echinochloa* (3)
	153 species	Poaceae (18)	*Kalanchoe* (3)
Jamaica	22 families	Fabaceae (8)	*Hedychium* (3)
	39 genera	Poaceae (7)	*Syzygium* (2)
	42 species	Myrtaceae (6)	
Puerto Rico	55 families	Poaceae (35)	*Cenchrus* (4)
	134 genera	Fabaceae (29)	*Cyperus* (4)
	176 species	Asteraceae (9)	*Eragrostis* (4)
Virgin Islands	47 families	Fabaceae (22)	*Eragrostis* (4)
	99 genera	Poaceae (21)	*Cenchrus* (3)
	122 species	Asteraceae (6)	*Tradescantia* (3)
St Lucia	17 families	Araceae (3)	*Tradescantia (2)*
	23 genera	Commelinaceae (3)	
	24 species	Fabaceae (2)	
St Martin	20 families	Poaceae (9)	*Sansevieria* (2)
	35 genera	Fabaceae (6)	
	36 species	Apocynaceae (2)	
Trinidad/Tobago	29 families	Fabaceae (13)	*Acacia* (2)
	53 genera	Poaceae (9)	*Paspalum* (2)
	56 species	Myrtaceae (3)	*Pterocarpus* (2)
All islands pooled	96 families	Fabaceae (88)	*Ipomoea* (10)
	348 genera	Poaceae (77)	*Paspalum* (7)
	516 species	Asteraceae (33)	*Eragrostis* (6)

Table S3. List of the 32 most widespread invasive plant species across nine islands in the West Indies. The invasive plant species included in this list are species occurring on five islands or more.

Species	*Family*	*Number of islands where occurring*	*Life-form*	*Life-history*
Leucaena leucocephala	Fabaceae	9	Tree	Perennial
Casuarina equisetifolia	Casuarinaceae	7	Tree	Perennial
Eichhornia crassipes	Pontederiaceae	7	Aquatic herb	Perennial
Megathyrsus maximus	Poaceae	7	Grass	Perennial
Melaleuca quinquenervia	Myrtaceae	7	Tree	Perennial
Ricinus communis	Euphorbiaceae	7	Shrub	Perennial
Spathodea campanulata	Bignoniaceae	7	Tree	Perennial
Terminalia catappa	Combretaceae	7	Tree	Perennial
Albizia lebbeck	Fabaceae	6	Tree	Perennial
Antigonon leptopus	Polygonaceae	6	Vine	Perennial
Calotropis procera	Apocynaceae	6	Shrub	Perennial
Echinochloa colona	Poaceae	6	Grass	Annual
Eleusine indica	Poaceae	6	Grass	Annual
Kalanchoe pinnata	Crassulaceae	6	Succulent	Perennial
Sansevieria hyacinthoides	Asparagaceae	6	Succulent	Perennial
Syngonium podophyllum	Araceae	6	Vine	Perennial
Urochloa mutica	Poaceae	6	Grass	Perennial
Albizia procera	Fabaceae	5	Tree	Perennial
Asystasia gangetica	Acanthaceae	5	Vine	Perennial
Bothriochloa pertusa	Poaceae	5	Grass	Perennial
Catharanthus roseus	Apocynaceae	5	Herb	Perennial
Cenchrus purpureus	Poaceae	5	Grass	Perennial
Cyanthillium cinereum	Asteraceae	5	Herb	Annual
Cyperus rotundus	Cyperaceae	5	Herb	Perennial
Dioscorea alata	Dioscoreaceae	5	Vine	Perennial
Epipremnum pinnatum	Araceae	5	Vine	Perennial
Jasminum fluminense	Oleaceae	5	Vine	Perennial
Melinis repens	Poaceae	5	Grass	Perennial
Schinus terebinthifolius	Anacardiaceae	5	Tree	Perennial
Syzygium jambos	Myrtaceae	5	Tree	Perennial
Tradescantia spathacea	Commelinaceae	5	Herb	Perennial
Tradescantia zebrina	Commelinaceae	5	Herb	Perennial

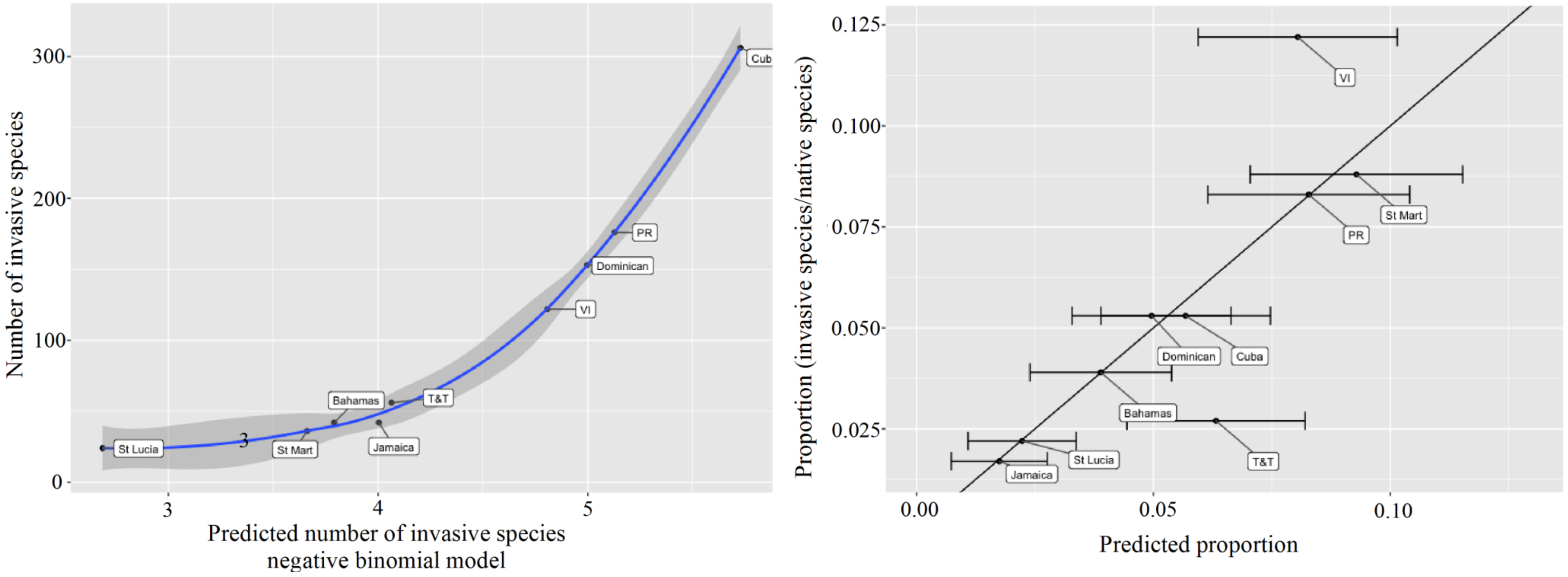

Figure S1. Relationship between (a) predicted negative binomial values of the model and the number of invasive plant species when all variables are included and (b) predicted beta regression values of the model and the ratio of invasive to native species. T&T = Trinidad and Tobago, VI = Virgin Islands, Dominican = Dominican Republic, PR = Puerto Rico, St Mart = St Martin.

Appendix S3. Coefficients estimated from the Generalized Linear Models for all 64 possible combinations of explanatory variables. Dependent variable: the number of invasive species. Coefficients were estimated with negative binomial error. Models are arranged by AICc values.

Model	Intercept	Elevation (m)	Island area (sq_Km)	Pop_density peop_sqKm	GDP (US$) percapita	Forest percentage	Paved roads (km)	df	logLik	AICc	ΔAIC	AICWeight
33	3.95889						0.00005194	3	-46.69831	104.1966	0	0.42201785
3	4.19452		0.00001459					3	-47.35864	105.5173	1.320668	0.21804766
2	3.98337	0.0004637						3	-49.08005	108.9601	4.763484	0.03899003
49	4.87352					-0.01888808	0.00004697	4	-45.70982	109.4196	5.223017	0.03098611
17	5.75763					-0.0244498		3	-49.39005	109.5801	5.383478	0.02859719
35	3.94297		0.00000833				0.00003347	4	-45.88517	109.7703	5.573713	0.02600246
11	3.2791		0.00002128		0.00003101			4	-46.17063	110.3413	6.144645	0.01954511
5	5.08308			-0.0016				3	-49.79168	110.3834	6.186733	0.0191381
41	3.46711				0.00001593		0.00006209	4	-46.28067	110.5613	6.364714	0.01750857
37	4.17409			-0.0007			0.00004895	4	-46.45995	110.9199	6.723284	0.01463485
9	5.08286				-0.00001867			3	-50.07725	110.9545	6.75788	0.01438387
34	3.84589	0.00012798					0.00004607	4	-46.49689	110.9938	6.797168	0.01410407
4	4.10256	0.00009386	0.0000131					4	-47.2962	112.5924	8.395774	0.00634179
7	4.09215		0.0000153	0.0003				4	-47.33305	112.6661	8.469484	0.00611232
19	4.07241		0.00001511			0.00233995		4	-47.3498	112.6996	8.502972	0.00601082
18	4.87743	0.00035988				-0.01657404		4	-48.58801	115.176	10.979398	0.00174255
6	4.29884	0.00040476		-0.0009				4	-48.82866	115.6573	11.460698	0.00136985
10	3.29548	0.00065668			0.0000181			4	-48.88053	115.7611	11.564435	0.00130061
21	5.74767			-0.0008		-0.01973934		4	-49.27891	116.5578	12.361208	0.00087323
25	5.82493				-0.0000069	-0.02248178		4	-49.34422	116.6884	12.491816	0.00081803
43	2.91387		0.00001516		0.00003355		0.00003754	5	-43.63943	117.2789	13.082241	0.00060892
13	5.12851			-0.0014	-0.00000424			4	-49.78065	117.5613	13.364686	0.00052872
12	2.28475	0.00043446	0.00001838		0.00005029			5	-44.69329	119.3866	15.18995	0.00021226

42	2.49386	0.00038689			0.00003667		0.00005634	5	-44.79096	119.5819	15.385294	0.00019251
45	3.55504			-0.0016	0.00003007		0.00006289	5	-45.18149	120.363	16.16636	0.00013027
57	4.42042				0.00001522	-0.0187488	0.00005417	5	-45.19066	120.3813	16.184705	0.00012908
51	4.58465		0.00000429			-0.01322944	0.00003963	5	-45.56256	121.1251	16.928496	0.00008899
53	4.90533			-0.0003		-0.01761021	0.00004641	5	-45.65575	121.3115	17.114882	0.00008107
50	4.76366	0.00005901				-0.01773114	0.00004473	5	-45.66234	121.3247	17.128054	0.00008054
39	4.00231		0.00000776	-0.0002			0.00003411	5	-45.86843	121.7369	17.540249	0.00006554
36	3.92855	0.00001729	0.00000805				0.00003326	5	-45.88194	121.7639	17.567258	0.00006466
27	2.97094		0.00002269		0.00003156	0.00555304		5	-46.09565	122.1913	17.994689	0.00005222
15	3.35216		0.0000208	-0.0004	0.00003272			5	-46.13353	122.2671	18.070436	0.00005028
38	4.04511	0.00010886		-0.0006			0.00004453	5	-46.31465	122.6293	18.432679	0.00004195
8	4.02325	0.00008829	0.00001377	0.00025				5	-47.27764	124.5553	20.358669	0.00001601
20	4.00006	0.00009177	0.00001357			0.002004		5	-47.28974	124.5795	20.382856	0.00001582
23	4.02254		0.00001557	0.00027		0.00150692		5	-47.32954	124.6591	20.462466	0.0000152
14	2.74357	0.00089529		-0.0024	0.00005512			5	-47.60922	125.2184	21.021813	0.00001149
26	4.16165	0.00055378			0.00002062	-0.01735		5	-48.28396	126.5679	22.371298	0.00000585
22	4.89907	0.00034958		-0.0005		-0.01403484		5	-48.53084	127.0617	22.865069	0.00000457
29	5.75919			-0.0007	-0.00000118	-0.01969299		5	-49.27802	128.556	24.359416	0.00000217
46	2.22496	0.00054268		-0.0022	0.00006431		0.00005542	6	-41.21429	136.4286	32.231968	0.00000004
44	2.2215	0.00032076	0.00001294		0.00004761		0.00003417	6	-42.06412	138.1282	33.931625	0.00000002
47	3.06211		0.00001311	-0.0011	0.00004041		0.00004229	6	-42.83514	139.6703	35.473652	0.00000001
59	3.05936		0.00001432		0.00003277	-0.00244845	0.00003814	6	-43.61889	141.2378	37.041166	0
16	2.23251	0.00056591	0.00001601	-0.0013	0.00006293			6	-43.97305	141.9461	37.749484	0
58	3.35749	0.00030741			0.00003135	-0.01256685	0.00005101	6	-44.22432	142.4486	38.25202	0
28	1.52606	0.00048327	0.00002114		0.00005477	0.01104465		6	-44.30034	142.6007	38.404066	0
61	4.24653			-0.0012	0.00002665	-0.01415727	0.00005708	6	-44.48591	142.9718	38.775193	0
55	4.62838		0.00000385	-0.0001		-0.01316363	0.00004014	6	-45.55129	145.1026	40.90595	0

52	4.57038	0.00001924	0.00000396			-0.01326725	0.00003944	6	-45.55835	145.1167	40.920082	0
54	4.80245	0.00005316		-0.0003		-0.01667651	0.00004445	6	-45.61765	145.2353	41.038685	0
40	3.98739	0.0000188	0.00000745	-0.0002			0.00003389	6	-45.86462	145.7292	41.532626	0
31	3.00595		0.00002232	-0.0005	0.00003396	0.00681932		6	-46.02434	146.0487	41.852051	0
24	3.96139	0.0000875	0.00001403	0.00022		0.00133953		6	-47.27487	148.5497	44.353113	0
30	3.22499	0.00080684		-0.0021	0.00005041	-0.00736643		6	-47.49768	148.9954	44.798732	0
48	2.00739	0.00048303	0.00000903	-0.0018	0.00006871		0.0000417	7	-38.23744	202.4749	98.278268	0
62	2.29373	0.00053406		-0.0022	0.00006357	-0.0009719	0.00005507	7	-41.20857	208.4171	104.220524	0
60	1.93881	0.00034189	0.00001417		0.00004981	0.00398787	0.000033	7	-41.9931	209.9862	105.789582	0
32	0.97098	0.00070171	0.00001962	-0.0018	0.00007499	0.01778102		7	-42.8045	211.609	107.412385	0
63	3.06979		0.00001307	-0.0011	0.00004036	-0.00013522	0.00004231	7	-42.83506	211.6701	107.473505	0
56	4.61426	0.00002087	0.00000348	-0.0002		-0.01320612	0.00003995	7	-45.54635	217.0927	112.896079	0
64	1.29678	0.00054563	0.00001184	-0.002	0.00007513	0.01059344	0.00003892	8	-37.02247	Inf	Inf	0

Appendix S4. Coefficients estimated from the Generalized Linear Models for all 64 possible combinations of explanatory variables. Dependent variable: ratio of invasive to native species. Coefficients were estimated with beta regression error. Models are arranged by AICc values.

Model	Intercept	Elevation (m)	Island area (sq_Km)	Pop_density peop_sqKm	GDP (US$) percapita	Forest percentage	Paved roads (km)	df	logLik	AICc	ΔAIC	AICWeight
9	-3.699972				0.0000338			3	21.68238	-32.564757	0	0.39221071
5	-3.186178			0.00114526				3	19.80233	-28.804651	3.7601059	0.0598443
11	-4.255686		0.00000942		0.00004768			4	23.17693	-28.353851	4.2109055	0.04776755
41	-4.240869				0.0000449		0.00002481	4	23.04103	-28.082063	4.4826944	0.04169795
2	-2.638735	-0.00015017						3	19.22452	-27.649042	4.915715	0.03358037
33	-2.855601						0.00000299	3	18.9873	-27.174595	5.390162	0.0264887
3	-2.84318		0.00000084					3	18.98544	-27.170886	5.3938709	0.02643962
17	-2.762944					-0.00131068		3	18.98103	-27.162057	5.4026998	0.02632316
10	-4.385845	0.00026899			0.00004758			4	22.3367	-26.673408	5.8913491	0.02061718
25	-3.491495				0.00003399	-0.00449479		4	21.75239	-25.504778	7.0599785	0.01149383
13	-3.700316			0.00009807	0.00003262			4	21.69104	-25.38207	7.1826867	0.01080983
7	-3.362862		0.00000435	0.00140706				4	20.06664	-22.133282	10.4314752	0.00212987
6	-3.000166	-0.00011738		0.00101302				4	19.95321	-21.906422	10.6583353	0.00190148
37	-3.316786			0.00127306			0.00000873	4	19.91799	-21.835978	10.7287789	0.00183567
21	-3.015419			0.00118777		-0.00388061		4	19.85926	-21.718517	10.8462404	0.00173096
34	-2.643373	-0.00030232					0.00001834	4	19.53896	-21.077928	11.4868293	0.00125657
4	-2.631416	-0.00022024	0.0000037					4	19.39295	-20.785896	11.778861	0.00108586
18	-2.183668	-0.00022019				-0.00785409		4	19.38603	-20.772061	11.7926958	0.00107837
35	-2.854892		0.00000039				0.00000208	4	18.98853	-19.97706	12.5876974	0.00072466
49	-2.833041					-0.00039962	0.00000263	4	18.98771	-19.975413	12.5893442	0.00072407
19	-2.813085		0.00000069			-0.00056941		4	18.9863	-19.972591	12.5921661	0.00072304

43	-4.497205		0.00000639		0.00005232		0.00001772	5	23.76327	-17.526539	15.0382179	0.00021282
12	-4.662828	0.00017873	0.00000855		0.00005561			5	23.50664	-17.013284	15.5514729	0.00016465
27	-4.889321		0.0000124		0.00005139	0.01004514		5	23.43708	-16.874162	15.6905949	0.00015359
42	-4.60453	0.00017512			0.0000521		0.00002119	5	23.26593	-16.531857	16.0329001	0.00012943
15	-4.246511		0.00000939	0.00012861	0.00004582			5	23.19583	-16.391667	16.1730899	0.00012066
57	-4.121903				0.00004607	-0.00331051	0.00002531	5	23.08793	-16.175865	16.3888919	0.00010832
45	-4.246245			-0.00006322	0.00004579		0.00002501	5	23.04504	-16.090085	16.4746717	0.00010377
14	-4.425739	0.00028436		-0.00017826	0.00005056			5	22.36397	-14.727942	17.836815	0.00005252
26	-4.522784	0.00028588			0.00004826	0.00207884		5	22.34935	-14.698692	17.8660651	0.00005176
29	-3.477548			0.00014484	0.0000323	-0.00482752		5	21.77115	-13.5423	19.0224567	0.00002903
8	-3.081246	-0.00023564	0.000007	0.00126416				5	20.56447	-11.128949	21.4358078	0.00000869
38	-3.034232	-0.00030237		0.00110867			0.00002198	5	20.48279	-10.965571	21.5991864	0.000008
22	-2.452227	-0.00020035		0.00101586		-0.00947115		5	20.20682	-10.413643	22.1511135	0.00000607
39	-3.374476		0.00000404	0.00141434			0.00000154	5	20.06887	-10.137738	22.4270189	0.00000529
23	-3.373126		0.0000044	0.00140782		0.00018867		5	20.06674	-10.133489	22.4312675	0.00000528
53	-3.218824			0.00128037		-0.00185551	0.00000757	5	19.92938	-9.858766	22.7059908	0.0000046
50	-2.334689	-0.00033861				-0.00522022	0.00001663	5	19.61277	-9.225543	23.339214	0.00000335
36	-2.640069	-0.00030367	0.00000103				0.00001611	5	19.54924	-9.098481	23.4662759	0.00000315
20	-2.305918	-0.00025108	0.00000266			-0.00565212		5	19.46541	-8.930819	23.6339378	0.00000289
51	-2.840831		0.00000036			-0.00024987	0.00000193	5	18.98868	-7.977358	24.5873987	0.0000018
28	-5.629208	0.00023249	0.00001216		0.0000621	0.01391026		6	24.02596	5.948085	38.5128417	0
44	-4.816312	0.00015067	0.00000625		0.00005855		0.00001531	6	23.96772	6.064558	38.6293148	0
59	-4.862297		0.00000866		0.00005327	0.00681443	0.00001507	6	23.8971	6.205798	38.7705547	0
47	-4.495108		0.0000064	0.00001684	0.00005206		0.00001765	6	23.7636	6.472796	39.0375533	0
16	-4.683362	0.00018574	0.00000853	-0.00006458	0.00005686			6	23.51096	6.978085	39.5428416	0
31	-4.890292		0.00001241	-0.0000017	0.00005142	0.01005714		6	23.43708	7.125832	39.6905889	0
46	-4.676091	0.00019965		-0.00023446	0.00005644		0.00002143	6	23.31678	7.366435	39.9311922	0

58	-4.556323	0.00016786			0.00005216	-0.00094964	0.00002153	6	23.26926	7.461488	40.0262445	0
61	-4.127305			-0.00003266	0.00004651	-0.00324202	0.00002541	6	23.089	7.821995	40.3867522	0
30	-4.667512	0.00031711		-0.00024544	0.00005279	0.00348071		6	22.39514	9.209716	41.7744727	0
40	-3.08423	-0.00031601	0.00000471	0.00126768			0.00001418	6	20.7261	12.547803	45.1125603	0
54	-2.571307	-0.00038171		0.00114108		-0.00802215	0.00002213	6	20.68559	12.628826	45.1935828	0
24	-2.788257	-0.00026202	0.00000603	0.00123203		-0.00487695		6	20.62594	12.748128	45.3128854	0
55	-3.397844		0.00000411	0.00141665		0.0004077	0.0000017	6	20.06933	13.861334	46.4260908	0
52	-2.334443	-0.00033865	-0.00000001			-0.00522466	0.00001665	6	19.61277	14.774456	47.3392134	0
60	-5.459537	0.00019739	0.00000953		0.00006153	0.01044382	0.00001042	7	24.26208	77.475836	110.0405931	0
32	-6.042792	0.00028308	0.00001329	-0.00041957	0.00007252	0.01782451		7	24.18023	77.639532	110.2042888	0
48	-4.866477	0.00016629	0.00000617	-0.00013689	0.00006131		0.00001562	7	23.98766	78.024687	110.5894444	0
63	-4.881834		0.00000872	-0.00004923	0.00005411	0.00704303	0.00001516	7	23.89984	78.200315	110.7650718	0
62	-4.678393	0.00020003		-0.00023518	0.00005645	0.00004233	0.00002142	7	23.31679	79.366423	111.9311801	0
56	-2.771154	-0.00035509	0.00000351	0.00123965		-0.00518157	0.00001546	7	20.79688	84.406242	116.9709992	0
64	-5.761351	0.00024294	0.00001039	-0.0003521	0.00006965	0.01328523	0.0000096	8	24.37971	Inf	Inf	0

Appendix S5. List of invasive plant species by island or island group. Species are ordered alphabetically by family name. Bah: Bahamas, DR=Dominican Republic, PR=Puerto Rico, VI= Virgin Islands, St. Lu= St Lucia, St. Ma= St Martin, T&T= Trinidad and Tobago.

**Family**	**Species**	**Author**	**Bah**	**Cuba**	**DR**	**Jam**	**PR**	**VI**	**St. Lu**	**St. Ma**	**T&T**
Acanthaceae	*Asystasia gangetica*	(L.) T. Anderson		✗	✗		✗	✗		✗	
Acanthaceae	*Barleria cristata*	L.					✗	✗			
Acanthaceae	*Barleria lupulina*	Lindl.			✗						
Acanthaceae	*Barleria prionitis*	L.					✗				
Acanthaceae	*Hemigraphis alternata*	W.Bull			✗						
Acanthaceae	*Nelsonia canescens*	(Lam.) Spreng.					✗				
Acanthaceae	*Odontonema cuspidatum*	(Nees) Kuntze		✗	✗						
Acanthaceae	*Odontonema nitidum*	Kuntze		✗							
Acanthaceae	*Pachystachys coccinea*	(Aubl.) Nees		✗							
Acanthaceae	*Ruellia macrophylla*	Vahl		✗							
Acanthaceae	*Ruellia simplex*	C.Wright	✗								
Acanthaceae	*Thunbergia alata*	Bojer ex Sims		✗	✗		✗	✗			
Acanthaceae	*Thunbergia fragrans*	Roxb.		✗			✗	✗			
Acanthaceae	*Thunbergia grandiflora*	Roxb.					✗	✗			
Agavaceae	*Yucca aloifolia*	L.		✗							
Amaranthaceae	*Achyranthes aspera*	L.		✗							
Amaranthaceae	*Alternanthera halimifolia*	(Lam.) Standl ex Pittier		✗							
Amaranthaceae	*Alternanthera philoxeroides*	(Mart.) Griseb.					✗				
Amaranthaceae	*Amaranthus dubius*	Mart ex Thell		✗							✗
Amaranthaceae	*Amaranthus spinosus*	L.		✗							
Amaranthaceae	*Chenopodium ambrosioides*	L.					✗	✗			
Amaranthaceae	*Chenopodium murale*	L.					✗	✗		✗	
Amaranthaceae	*Gomphrena globosa*	L.		✗							

Anacardiaceae	*Mangifera indica*	L.					✗	✗		✗	✗
Anacardiaceae	*Rhus succedanea*	L.		✗							
Anacardiaceae	*Schinus terebinthifolius*	Raddi	✗	✗		✗	✗	✗			
Annonaceae	*Polyalthia suberosa*	(Roxb.) Thwaites		✗							
Apiaceae	*Anethum graveolens*	L.			✗						
Apiaceae	*Conium maculatum*	L.			✗						
Apiaceae	*Foeniculum vulgare*	Mill.		✗							
Apocynaceae	*Allamanda cathartica*	L.					✗	✗			
Apocynaceae	*Calotropis procera*	(Aiton) W.T. Aiton		✗	✗		✗	✗		✗	✗
Apocynaceae	*Catharanthus roseus*	(L.) G. Don		✗	✗		✗	✗		✗	
Apocynaceae	*Cryptostegia madagascariensis*	Bojer ex Decne.					✗	✗	✗		
Apocynaceae	*Rauvolfia caffra*	Sond.		✗							
Apocynaceae	*Trachelospermum jasminoides*	(Lindl.) Lem	✗								
Araceae	*Alocasia macrorrhizos*	(L.) G. Don		✗		✗	✗				
Araceae	*Alocasia plumbea*	Van Houtte					✗				
Araceae	*Caladium bicolor*	(Aiton) Vent.									✗
Araceae	*Colocasia esculenta*	(L.) Schott					✗				
Araceae	*Dieffenbachia seguine*	(Jacq.) Schott		✗							
Araceae	*Epipremnum pinnatum*	(L.) Engl.		✗	✗		✗	✗	✗		
Araceae	*Pistia stratiotes*	L.		✗					✗		
Araceae	*Syngonium podophyllum*	Schott	✗	✗	✗		✗	✗	✗		
Araceae	*Xanthosoma sagittifolium*	(L.) Schott					✗	✗			
Araliaceae	*Schefflera actinophylla*	(Endl.) Harms	✗	✗			✗	✗			
Arecaceae	*Chamaedorea elegans*	Mart.		✗							
Arecaceae	*Cocos nucifera*	L.					✗	✗			
Arecaceae	*Dypsis lutescens*	(H. Wendl.) Beentje & J. Dransf.		✗							
Arecaceae	*Ptychosperma elegans*	(R.Br) Blume		✗							
Aristolochiaceae	*Aristolachia elegans*	Mast.		✗							

Aristolochiaceae	*Aristolachia ringens*	Vahl		✗							
Asclepiadaceae	*Cryptostegia grandiflora*	(Roxb.) R.Br.		✗	✗					✗	
Asparagaceae	*Agave angustifolia*	Tel.		✗							
Asparagaceae	*Asparagus aethiopicus*	L.	✗	✗							
Asparagaceae	*Asparagus setaceus*	(Kunth) Jessop			✗						
Asparagaceae	*Sansevieria hyacinthoides*	(L.) Druce	✗	✗	✗		✗	✗		✗	
Asparagaceae	*Sanseviera trifasciata*	Prain		✗			✗	✗		✗	
Asteraceae	*Achillea millefolium*	L.			✗						
Asteraceae	*Ageratum conyzoides*	L.		✗							
Asteraceae	*Ageratum houstonianum*	Mill.		✗							
Asteraceae	*Ambrosia peruviana*	Willd.		✗							
Asteraceae	*Centratherum punctatum*	Cass.			✗		✗	✗			
Asteraceae	*Chromolaena odorata*	(L.) R.M. King & H. Rob.		✗							
Asteraceae	*Cirsium mexicanum*	DC.		✗			✗				
Asteraceae	*Conyza canadensis*	(L.) Cronquist		✗							
Asteraceae	*Crassocephalum crepidioides*	(Benth.) S. Moore			✗		✗				
Asteraceae	*Cyanthillium cinereum*	(L.) H. Rob.		✗	✗		✗	✗			✗
Asteraceae	*Emilia coccinea*	(Sims) G.Don			✗						
Asteraceae	*Emilia fosbergii*	Nicolson			✗		✗	✗			
Asteraceae	*Emilia sonchifolia*	(L.) DC.			✗		✗	✗			✗
Asteraceae	*Erechtites hieracifolia*	(L.) Raf.			✗						
Asteraceae	*Erechtites valerianifolia*	(L.) Raf.			✗						
Asteraceae	*Gaillardia pulchella*	Foug.			✗						
Asteraceae	*Galinsoga quadriradiata*	Ruiz & Pav.					✗				
Asteraceae	*Gerbera jamesonii*	Bolus ex Hook.f.			✗						
Asteraceae	*Helenium amarum*	(Raf.) H.Rock		✗							
Asteraceae	*Helenium quadridentatum*	Labill.		✗							
Asteraceae	*Launaea intybacea*	(Jacq.) Beauverd		✗	✗	✗					

Asteraceae	*Leucanthemum vulgare*	Lam.			✗						
Asteraceae	*Parthenium hysterophorus*	L.		✗						✗	
Asteraceae	*Pseudelephantopus spicatus*	(Juss.) Rohr		✗							
Asteraceae	*Solidago sempervirens*	Michx.			✗						
Asteraceae	*Sonchus asper*	(L.) Hill			✗						
Asteraceae	*Sonchus oleraceus*	L.		✗			✗	✗			
Asteraceae	*Sphagneticola trilobata*	(L.) Pruski	✗	✗			✗	✗			
Asteraceae	*Tagetes erecta*	L.		✗							
Asteraceae	*Tithonia diversifolia*	(Hemsl.) A. Gray		✗	✗						
Asteraceae	*Viguiera dentata*	(Cav.) Spreng.		✗							
Asteraceae	*Xanthium strumarium*	L.			✗						
Asteraceae	*Youngia japonica*	(L.) DC.		✗	✗						
Balsaminaceae	*Impatiens balsamina*	L.		✗			✗	✗			
Balsaminaceae	*Impatiens walleriana*	Hook.f.			✗						
Begoniaceae	*Begonia nelumbiifolia*	Schltdl. & Cham.		✗	✗						
Bignoniaceae	*Macfadyena ungis-cati*	(L.) A.H. Gentry	✗	✗							
Bignoniaceae	*Spathodea campanulata*	P. Beauv.	✗	✗	✗	✗	✗	✗	✗		
Bignoniaceae	*Tabebuia rosea*	(Bertol.) DC.			✗		✗	✗			✗
Bignoniaceae	*Tecoma capensis*	(Thunb.) Spach		✗							
Boraginaceae	*Cordia obliqua*	Willd.		✗			✗	✗	✗		
Boraginaceae	*Cynoglossum amabile*	Stapf & J.R.Drumm			✗						
Boraginaceae	*Heliotropium indicum*	L.		✗							
Brassicaceae	*Brassica juncea*	(L.) Czern.		✗	✗						
Brassicaceae	*Brassica rapa*	DC		✗	✗						
Brassicaceae	*Lepidium virginicum*	L.		✗							
Bromeliaceae	*Bromelia pinguin*	L.		✗							
Cactaceae	*Hylocereus undatus*	Britton & Rose		✗							
Cactaceae	*Nopalea cochenillifera*	(L.) Salm-Dyck		✗							

Cactaceae	*Opuntia stricta*	(Haw.) Haw. Var stricta		✗							
Caprifoliaceae	*Lonicera confusa*	DC		✗							
Caprifoliaceae	*Lonicera japonica*	Thunb.			✗		✗	✗			
Caryophyllaceae	*Drymaria cordata*	(L.) Willd. ex Roem. Schult.					✗	✗			✗
Casuarinaceae	*Casuarina cunninghamiana*	Miq.		✗							
Casuarinaceae	*Casuarina equisetifolia*	L.	✗	✗	✗	✗	✗	✗			✗
Casuarinaceae	*Casuarina glauca*	Sieb.	✗	✗							
Ceratophyllaceae	*Ceratophyllum demersum*	L.		✗							
Cleomaceae	*Arivela viscosa*	(L.) Raf.			✗		✗	✗		✗	
Cleomaceae	*Cleome gynandra*	L.		✗	✗					✗	
Cleomaceae	*Cleome rutidosperma*	DC.									✗
Cleomaceae	*Gynandropsis gynandra*	(L.) Briq.			✗		✗	✗			
Clusiaceae	*Calophyllum inophyllum*	L.		✗							
Clusiaceae	*Mammea americana*	L.		✗							
Combretaceae	*Quisqualis indica*	L.		✗							
Combretaceae	*Terminalia catappa*	L.	✗	✗	✗	✗	✗	✗			✗
Commelinaceae	*Callisia fragrans*	(Lindl.) Woodson							✗		
Commelinaceae	*Callisia repens*	(Jacq.) L.		✗							
Commelinaceae	*Commelina diffusa*	Burm. f.		✗							✗
Commelinaceae	*Murdannia nudiflora*	(L.) Brenan									✗
Commelinaceae	*Tradescantia pallida*	(Rose) D.R. Hunt		✗			✗	✗			
Commelinaceae	*Tradescantia spathacea*	Sw.	✗	✗			✗	✗	✗		
Commelinaceae	*Tradescantia zebrina*	Heynh. ex Bosse		✗	✗		✗	✗	✗		
Convolvulaceae	*Argyreia nervosa*	(Burm.f.) Bojer		✗							
Convolvulaceae	*Ipomoea alba*	L.		✗							
Convolvulaceae	*Ipomoea aquatica*	Forssk.		✗	✗						
Convolvulaceae	*Ipomoea fistulosa*	Mart. ex Choisy		✗							
Convolvulaceae	*Ipomoea hederifolia*	L.		✗							

Convolvulaceae	*Ipomoea indica*	(Burm.) Merr.	✗								
Convolvulaceae	*Ipomoea nil*	(L.) Roth.		✗							
Convolvulaceae	*Ipomoea obscura*	(L.) Ker Gawl.		✗							
Convolvulaceae	*Ipomoea ochracea*	(Lindl.) G. Don					✗	✗			
Convolvulaceae	*Ipomoea purpurea*	(L.) Roth.	✗	✗							
Convolvulaceae	*Ipomoea quamoclit*	L.		✗							
Convolvulaceae	*Merremia aegyptia*	(L.) Urb.		✗							
Convolvulaceae	*Merremia cissoides*	(Lam.) Hallier f.		✗							
Convolvulaceae	*Merremia tuberosa*	(L.) Rendle		✗		✗					
Convolvulaceae	*Merremia umbellata*	(L.) Hallier f.		✗							
Convolvulaceae	*Turbina corymbosa*	(L.) Raf.		✗							
Crassulaceae	*Kalanchoe blossfeldiana*	Poelln.			✗						
Crassulaceae	*Kalanchoe daigremontiana*	Raym.-Hamet & Perrier		✗			✗				
Crassulaceae	*Kalanchoe delagoensis*	Ecklon & Séller		✗	✗						
Crassulaceae	*Kalanchoe integra*	(Medik.) Kuntze		✗							
Crassulaceae	*Kalanchoe pinnata*	(Lam.) Pers.		✗	✗		✗	✗	✗	✗	
Cucurbitaceae	*Coccinia grandis*	(L.) Voigt			✗		✗	✗	✗		
Cucurbitaceae	*Cucumis dipsaceus*	Ehrenb.		✗	✗						
Cucurbitaceae	*Elaterium carthagenense*	Jacq.		✗							
Cucurbitaceae	*Luffa acutangula*	(L.) Roxb.		✗							
Cucurbitaceae	*Momordica charantia*	L.		✗			✗	✗			✗
Cucurbitaceae	*Sechium edule*	(Jacq.) Sw.		✗			✗				
Cupressaceae	*Cupressus arizonica*	Greene			✗						
Cupressaceae	*Cupressus sempervirens*	L.		✗							
Cyclanthaceae	*Carludovica palmata*	Ruiz & Pav.		✗							
Cyperaceae	*Cyperus difformis*	L.					✗				
Cyperaceae	*Cyperus esculentus*	L.		✗	✗		✗	✗			
Cyperaceae	*Cyperus imbricatus*	Retz.					✗				

Cyperaceae	*Cyperus involucratus*	Kuk		✗							
Cyperaceae	*Cyperus rotundus*	L.			✗	✗	✗	✗			✗
Cyperaceae	*Fimbristylis littoralis*	Gaudich.					✗				✗
Cyperaceae	*Kyllinga nemoralis*	(J.R. Forst. & G. Forst.) Dandy ex Hutch. & Dalziel					✗				
Dioscoreaceae	*Dioscorea alata*	L.	✗	✗	✗		✗	✗			
Dioscoreaceae	*Dioscore bulbifera*	L.	✗	✗			✗				
Elaeagnaceae	*Elaeagnus umbellata*	Thunb.			✗						
Euphorbiaceae	*Aleurites moluccanus*	(L.) Willd.			✗						✗
Euphorbiaceae	*Codiaeum variegatum*	(L.) Rumph. ex A. Juss.					✗	✗			
Euphorbiaceae	*Croton argenteus*	L.		✗							
Euphorbiaceae	*Euphorbia lactea*	Haw.		✗							
Euphorbiaceae	*Euphorbia tirucalli*	L.		✗							
Euphorbiaceae	*Euphorbia trigona*	Haw.		✗							
Euphorbiaceae	*Garcia nutans*	Vahl		✗							
Euphorbiaceae	*Jatropha curcas*	L.					✗	✗			✗
Euphorbiaceae	*Reutealis trisperma*	(Blanco) Airy Shaw			✗						
Euphorbiaceae	*Ricinus communis*	L.	✗	✗	✗	✗	✗	✗		✗	
Euphorbiaceae	*Synadenium grantii*	Hook.f.			✗						
Euphorbiaceae	*Vernicia fordii*	(Hemsl.) Airy Shaw			✗						
Fabaceae	*Abrus precatorius*	L.	✗	✗			✗	✗			
Fabaceae	*Acacia angustissima*	(Mill.) Kuntze			✗						
Fabaceae	*Acacia auriculiformis*	A. Cunn. ex Benth									✗
Fabaceae	*Acacia longifolia*	(Andrews) Willd.			✗						
Fabaceae	*Acacia mangium*	Willd.			✗						✗
Fabaceae	*Acacia mearnsii*	De Wild				✗					
Fabaceae	*Adenanthera pavonina*	L.			✗	✗	✗	✗			
Fabaceae	*Aeschynomene americana*	L.		✗							

Fabaceae	*Albizia lebbeck*	(L.) Benth.	✗	✗	✗		✗	✗		✗	
Fabaceae	*Albizia procera*	(Roxb.) Benth.		✗	✗		✗	✗			✗
Fabaceae	*Alysicarpus vaginalis*	(L.) DC.			✗		✗	✗			
Fabaceae	*Bauhinia monandra*	Kurz			✗						
Fabaceae	*Bauhinia purpurea*	L.		✗							
Fabaceae	*Bauhinia tomentosa*	L.		✗							
Fabaceae	*Bauhinia variegata*	L.	✗	✗							
Fabaceae	*Caesalpinia bonduc*	(L.) Roxb.		✗						✗	
Fabaceae	*Caesalpinia decapetala*	(Roth) Alst.					✗				
Fabaceae	*Caesalpinia pulcherrima*	(L.) Sw.					✗	✗			
Fabaceae	*Caesalpinia violacea*	(Mill.) Standl.		✗							
Fabaceae	*Calliandra houstoniana*	(Mill.) Standl.			✗						
Fabaceae	*Calopogonium caeruleum*	(Benth.) C. Wright		✗			✗				
Fabaceae	*Calopogonium mucunoides*	Desv.					✗				
Fabaceae	*Canavalia ensiformis*	(L.) DC.					✗	✗			
Fabaceae	*Cassia javanica*	L.			✗						
Fabaceae	*Clitoria ternatea*	L.								✗	
Fabaceae	*Copaifera officinalis*	L.			✗						
Fabaceae	*Coronilla varia*	L.			✗						
Fabaceae	*Crotalaria maypurensis*	Kunth		✗							
Fabaceae	*Crotalaria retusa*	L.		✗			✗	✗			
Fabaceae	*Crotalaria spectabilis*	Roth		✗							
Fabaceae	*Crotalaria verrucosa*	L.					✗	✗			
Fabaceae	*Cytisus scoparius*	(L.) Link			✗						
Fabaceae	*Dalea scandens*	(Mill.) R.T.Clausen		✗							
Fabaceae	*Delonix regia*	(Bojer ex Hook.) Raf.	✗	✗	✗					✗	
Fabaceae	*Dichrostachys cinerea*	(L.) Wight & Arn.		✗	✗	✗					
Fabaceae	*Entada gigas*	(L.) Faxc. & Rendle		✗							

Fabaceae	*Erythrina berteroana*	Urb.		✗							
Fabaceae	*Erythrina poeppigiana*	(Walp.) O.F. Cook		✗			✗				
Fabaceae	*Erythrina variegata*	L.					✗	✗			
Fabaceae	*Falcataria moluccana*	(Miq.) Barneby & J.W. Grimes		✗							
Fabaceae	*Flemingia lineata*	(L.) Aiton		✗							
Fabaceae	*Flemingia strobilifera*	(L.) W.T.Aiton			✗	✗					
Fabaceae	*Gliricidia sepium*	(Jacq.) Kunth									✗
Fabaceae	*Haematoxylum campechianum*	L.	✗			✗					
Fabaceae	*Indigofera jamaicensis*	Spreng.			✗						
Fabaceae	*Indigofera spicata*	Forssk.					✗	✗			
Fabaceae	*Indigofera tinctoria*	L.		✗						✗	
Fabaceae	*Inga punctata*	Willd.		✗							
Fabaceae	*Lablab purpureus*	Medik		✗							
Fabaceae	*Lathyrus odoratus*	L.			✗						
Fabaceae	*Leucaena leucocephala*	(Lam.) De Wit	✗	✗	✗	✗	✗	✗	✗	✗	✗
Fabaceae	*Lupinus angustifolius*	L.			✗						
Fabaceae	*Lupinus plattensis*	S.Watson			✗						
Fabaceae	*Macroptilium atropurpureum*	(DC.) Urb.			✗						
Fabaceae	*Medicago lupulina*	L.			✗						
Fabaceae	*Medicago polymorpha*	L.			✗						
Fabaceae	*Melilotus officinalis*	(Medik.) H.Ohashi & Tateishi			✗						
Fabaceae	*Mimosa casta*	L.					✗				
Fabaceae	*Mimosa pigra*	L.		✗	✗		✗		✗		
Fabaceae	*Mucuna pruriens*	(L.) DC.	✗								
Fabaceae	*Myroxylon balsamum*	(L.) Harms									✗
Fabaceae	*Parkinsonia aculeata*	L.		✗	✗						
Fabaceae	*Phaseolus lunatus*	L.		✗			✗	✗			
Fabaceae	*Pithecellobium dulce*	(Roxb.) Benth.		✗	✗		✗	✗			

Fabaceae	*Prosopis juliflora*	(Sw.) DC.		✗			✗	✗			✗
Fabaceae	*Prosopis pallida*	(Humb. & Bonpl. ex Willd.) Kunth					✗	✗			
Fabaceae	*Pterocarpus indicus*	Willd.									✗
Fabaceae	*Pterocarpus macrocarpus*	Kurz									✗
Fabaceae	*Pueraria phaseoloides*	(Roxb.) Benth.					✗	✗			✗
Fabaceae	*Schizolobium parahyba*	(Vieill.) S.F. Blake		✗							
Fabaceae	*Senna alata*	(L.) Roxb.		✗							
Fabaceae	*Senna multijuga*	(Viv.) H.S. Irwin & Barneby									✗
Fabaceae	*Senna occidentalis*	(L.) Link		✗							
Fabaceae	*Senna siamea*	(DC.) H.S. Irwin & Barneby			✗		✗	✗			
Fabaceae	*Senna spectabilis*	(DC.)		✗	✗						
Fabaceae	*Sesbania bispinosa*	(Jacq.) W. Wright		✗							✗
Fabaceae	*Sesbania sericea*	(Willd.) Link		✗			✗	✗			
Fabaceae	*Sesbania sesban*	(L.) Merr.		✗			✗	✗			
Fabaceae	*Spartium junceum*	L.			✗						
Fabaceae	*Tamarindus indica*	L.					✗	✗			
Fabaceae	*Tephrosia candida*	DC.									✗
Fabaceae	*Trifolium dubium*	Sibth.			✗						
Fabaceae	*Trifolium pratense*	L.			✗						
Fabaceae	*Trifolium repens*	L.			✗						
Fabaceae	*Ulex europaeus*	L.				✗					
Fabaceae	*Vachellia farnesiana*	(L.) Wight & Arn.		✗		✗	✗	✗			
Fabaceae	*Vachellia macracantha*	Humb. & Bonpl. Ex Willd		✗							
Fabaceae	*Vachellia nilotica*	(L.) P.J.H. Hurter & Mabb.					✗				
Goodeniaceae	*Scaevola sericea*	Vahl		✗							
Goodeniaceae	*Scaevola taccada*	(Gaertn.) Roxb.	✗				✗	✗	✗		
Haloragaceae	*Myriophyllum aquaticum*	(Vell.) Verdc.			✗						
Haloragaceae	*Myriophyllum pinnatum*	(Walter) Britton, Sterns & Poggenb		✗							

Hydrocharitaceae	*Egeria densa*	Planch.		✗			✗				
Hydrocharitaceae	*Elodea canadensis*	Michx.		✗							
Hydrocharitaceae	*Halophila stipulacea*	(Forssk.) Asch.							✗		
Hydrocharitaceae	*Hydrilla verticillata*	(L.f) Royle		✗	✗	✗					
Iridaceae	*Crocosmia crocosmiiflora*	(Lemoine) N.E.Br.			✗						
Iridaceae	*Neomarica caerulea*	(Ker Gawl.) Sprague		✗							
Iridaceae	*Trimezia steyermarkii*	R.C. Foster		✗							
Lamiaceae	*Clerodendrum chinense*	(Osbeck) Mabb.		✗	✗		✗	✗			
Lamiaceae	*Clerodendrum quadriloculare*	(Blanco) Merr.			✗		✗				
Lamiaceae	*Clerodendrum speciosissimum*	C. Morren		✗							
Lamiaceae	*Gmelina arborea*	Roxb.			✗						
Lamiaceae	*Hyptis brevipes*	Willd.		✗							
Lamiaceae	*Hyptis spicigera*	Lam.		✗							
Lamiaceae	*Leonotis nepetifolia*	(L.) R. Br.		✗			✗	✗			
Lamiaceae	*Leonurus japonicus*	Houtt.					✗	✗			
Lamiaceae	*Leonurus sibiricus*	L.		✗							
Lamiaceae	*Plectranthus scutellarioides*	(L.) R. Br.		✗							
Lamiaceae	*Plectranthus amboinicus*	(Lour.) Spreng		✗							
Lamiaceae	*Prunella vulgaris*	L.			✗						
Lamiaceae	*Tectona grandis*	L. f.									✗
Lamiaceae	*Vitex trifolia*	L.		✗							
Lauraceae	*Cassytha filiformis*	L.		✗							
Lauraceae	*Cinnamomum camphora*	(L.) J.Presl			✗						
Lauraceae	*Persea americana*	Mill.									✗
Lecythidaceae	*Barringtonia asiatica*	(L.) Kurz			✗						
Lemnaceae	*Lemna aequinoctialis*	Welw.		✗							
Lemnaceae	*Lemna perpusilla*	Torr.		✗							
Linderniaceae	*Lindernia crustacea*	(L.) F.Muell.									✗

Lythraceae	*Cuphea hyssopifolia*	Kunth		✗	✗						
Lythraceae	*Lagerstroemia indica*	L.					✗	✗			
Lythraceae	*Lagerstroemia speciosa*	(L.) Pers.					✗	✗			
Malpighiaceae	*Galphimia glauca*	Cav.		✗							
Malvaceae	*Abutilon hirtum*	(Lam.) Sweet		✗							
Malvaceae	*Abutilon indicum*	(L.) Sweet		✗							
Malvaceae	*Dombeya wallichii*	(Lindl.) K. Schum		✗							
Malvaceae	*Gossypium barbadense*	L.		✗						✗	
Malvaceae	*Hibiscus elatus*	Sw.									✗
Malvaceae	*Malva pusilla*	Sm.			✗						
Malvaceae	*Pachira aquatica*	Aubl.			✗						
Malvaceae	*Pachira insignis*	(Sw.) Savigny			✗						
Malvaceae	*Sida linifolia*	Cav.		✗							
Malvaceae	*Sida repens*	Dombey ex Cav.					✗	✗			
Malvaceae	*Sida ulmifolia*	Mill.		✗							
Malvaceae	*Sterculia apetala*	(Jacq.) H. Karst.					✗				✗
Malvaceae	*Talipariti tiliaceum*	(L.) Fryxell		✗							
Malvaceae	*Thespesia populnea*	(L.) Sol. exCorrea	✗								
Malvaceae	*Urena lobata*	L.		✗							
Malvaceae	*Urena sinuata*	L.		✗							
Melastomataceae	*Heterotis rotundifolia*	(Sm.) Triana					✗				
Melastomataceae	*Miconia calvescens*	DC.			✗	✗					
Meliaceae	*Azadirachta indica*	A. Juss			✗					✗	✗
Meliaceae	*Melia azedarach*	L.			✗		✗	✗			
Moraceae	*Artocarpus altilis*	(Parkinson) Fosberg					✗	✗			✗
Moraceae	*Castilla elastica*	Cerv.		✗	✗		✗	✗			
Moraceae	*Ficus benghalensis*	L.	✗								
Moraceae	*Ficus pumila*	L.		✗							

Moringaceae	*Moringa oleifera*	Lam.		✗						✗	
Muntingiaceae	*Muntingia calabura*	L.					✗				
Myrtaceae	*Eucalyptus camaldulensis*	Dehnh.				✗					
Myrtaceae	*Eucalyptus citriodora*	Hook.		✗							
Myrtaceae	*Eucalyptus robusta*	Sm.					✗				
Myrtaceae	*Eucalyptus cinerea*	F.Muell. ex Benth.			✗						
Myrtaceae	*Eugenia uniflora*	L.	✗		✗	✗					
Myrtaceae	*Melaleuca quinquenervia*	(Cav.) S.T. Blake	✗	✗	✗	✗	✗	✗			✗
Myrtaceae	*Pimenta racemosa*	(Mill.) J.W. Moore	✗								
Myrtaceae	*Psidium cattleianum*	Afzel. Ex Sabine				✗					
Myrtaceae	*Psidium guajava*	L.		✗						✗	✗
Myrtaceae	*Syzygium cumini*	(L.) Skeels				✗					✗
Myrtaceae	*Syzygium jambos*	(L.) Alston		✗	✗	✗	✗	✗			
Myrtaceae	*Syzygium malaccense*	(L.) Merr. & L.M.Perry			✗						
Nyctaginaceae	*Mirabilis jalapa*	L.			✗		✗	✗			
Oleaceae	*Fraxinus americana*	L.		✗							
Oleaceae	*Jasminum fluminense*	Vell.	✗	✗			✗	✗		✗	
Oleaceae	*Jasminum multiflorum*	(Burm. f.) Andrews					✗	✗			
Oleaceae	*Jasminum sambac*	(L.) Aiton	✗	✗							
Orchidaceae	*Epidendrum radicans*	Pav.ex Lindl.		✗							
Orchidaceae	*Oeceoclades maculata*	(Lindl.) Lindl.		✗			✗	✗	✗		
Orchidaceae	*Phaius tankervilleae*	(Banks L'Hér.) Blume		✗							
Orchidaceae	*Spathoglottis plicata*	Blume		✗			✗		✗		
Oxalidaceae	*Averrhoa carambola*	L.									✗
Papaveraceae	*Argemone mexicana*	L.		✗							
Passifloraceae	*Passiflora edulis*	Sims					✗	✗			
Passifloraceae	*Turnera ulmifolia*	L.					✗	✗			
Paulowniaceae	*Paulownia tomentosa*	Steud.			✗						

Pedaliaceae	*Martynia annua*	L.		✗							
Phyllanthaceae	*Breynia disticha*	J.R. Forst & G. Forst		✗	✗						
Phyllanthaceae	*Phyllanthus urinaria*	L.					✗				
Phytolaccaceae	*Agdestis clematidea*	Moc. & Sessé ex DC		✗							
Pinaceae	*Pinus caribaea*	Morelet			✗		✗				
Piperaceae	*Piper auritum*	Kunth		✗							
Pittosporaceae	*Pittosporum undulatum*	Vent.				✗					
Plantaginaceae	*Plantago lanceolata*	L.		✗							
Plantaginaceae	*Plantago major*	L.		✗							
Plantaginaceae	*Russelia equisetiformis*	Schltdl. & Cham.		✗							
Plumbaginaceae	*Plumbago auriculata*	Lam.					✗	✗			
Poaceae	*Aristida ternipes*	Cav.		✗							
Poaceae	*Arundo donax*	L.		✗	✗		✗				
Poaceae	*Avena fatua*	L.			✗						
Poaceae	*Bambusa bambos*	(L.) Voss		✗							
Poaceae	*Bambusa vulgaris*	Schrad. ex H.L. Wendl.		✗		✗	✗				✗
Poaceae	*Bothriochloa bladhii*	(Retz.) S.T. Blake		✗							
Poaceae	*Bothriochloa pertusa*	(L.) A. Camus		✗	✗		✗	✗		✗	
Poaceae	*Brachiaria decumbens*	Stapf.				✗					
Poaceae	*Brachiaria eruciformis*	(Sm.) Griseb.		✗							
Poaceae	*Cenchrus ciliaris*	L.		✗			✗	✗			
Poaceae	*Cenchrus polystachios*	(L.) Morrone		✗			✗				
Poaceae	*Cenchrus purpureus*	(Schumach.) Morrone	✗	✗			✗	✗		✗	
Poaceae	*Cenchrus setaceus*	(Forssk.) Morrone					✗	✗			
Poaceae	*Chloris barbata*	Sw.		✗							
Poaceae	*Chloris virgata*	Sw.		✗							
Poaceae	*Coix lacryma-jobi*	L.				✗	✗	✗			
Poaceae	*Cymbopogon citratus*	(DC.) Stapf							✗		

Poaceae	*Cynodon dactylon*	(L.) Pers.		✗			✗	✗			✗
Poaceae	*Cynodon nlemfuensis*	Vanderhyst		✗			✗				
Poaceae	*Cynodon plectostachyus*	(K. Schum.) Pilg.		✗							
Poaceae	*Dactyloctenium aegyptium*	(L.) Willd.					✗	✗		✗	
Poaceae	*Dendrocalamus strictus*	(Robx.) Nees		✗							
Poaceae	*Dichanthium annulatum*	(Forssk.) Stapf		✗			✗				
Poaceae	*Dichanthium caricosum*	(L.) A. Camus		✗							
Poaceae	*Digitaria ciliaris*	(Retz.) Koeler		✗			✗	✗			
Poaceae	*Digitaria eriantha*	Steud.		✗	✗						
Poaceae	*Digitaria insularis*	(L.) Fedde		✗							
Poaceae	*Digitaria sanguinalis*	(L.) Scop.					✗	✗			
Poaceae	*Echinochloa colona*	(L.) Link		✗	✗		✗	✗		✗	✗
Poaceae	*Echinochloa crus-galli*	(L.) P. Beauv.		✗	✗						
Poaceae	*Echinochloa crus-pavonis*	(Kunth) Schult.		✗	✗						
Poaceae	*Eleusine indica*	(L.) Gaertn.		✗	✗		✗	✗		✗	✗
Poaceae	*Eragrostis atrovirens*	(Desf.) Trin ex Steud.		✗							
Poaceae	*Eragrostis ciliaris*	(L.) R. Br.		✗			✗	✗		✗	
Poaceae	*Eragrostis japonica*	(Thunb.) Trin.		✗							
Poaceae	*Eragrostis pilosa*	(L.) P. Beauv.					✗	✗			
Poaceae	*Eragrostis tenella*	(L.) P. Beauv. ex Roem. & Schult.		✗			✗	✗			
Poaceae	*Eragrostis unioloides*	(Retz.) Nees ex Steud.					✗	✗			
Poaceae	*Eremochloa ophiuroides*	(Munro) Hack.					✗				
Poaceae	*Heteropogon contortus*	(L.) P. Beaux ex Roem. & Schult		✗							
Poaceae	*Hyparrhenia rufa*	(Nees) Stapf		✗	✗		✗				
Poaceae	*Ischaemum rugosum*	Salisb.		✗	✗						
Poaceae	*Leptochloa fusca*	(J. Pres;) N.W. Snow		✗							
Poaceae	*Leptochloa mucronata*	(Michx.) Kunth		✗							
Poaceae	*Leptochloa nealleyi*	Vasey		✗							

Poaceae	*Lolium perenne*	L.			✗						
Poaceae	*Megathyrsus maximus*	(Jacq.) B.K. Simon & S.W.L. Jacobs	✗	✗	✗	✗	✗			✗	✗
Poaceae	*Melinis minutiflora*	P. Beauv.		✗	✗	✗	✗				
Poaceae	*Melinis repens*	(Willd.) Zizka		✗	✗		✗	✗		✗	
Poaceae	*Panicum repens*	L.	✗				✗	✗			
Poaceae	*Paspalum densum*	Poir		✗							
Poaceae	*Paspalum fasciculatum*	Willd. ex Flüggé					✗				✗
Poaceae	*Paspalum millegrana*	Schrad.		✗							
Poaceae	*Paspalum notatum*	Fluggé		✗							
Poaceae	*Paspalum paniculatum*	L.		✗							✗
Poaceae	*Paspalum urvillei*	Steud.					✗				
Poaceae	*Paspalum virgatum*	L.		✗							
Poaceae	*Phyllostachys aurea*	Rivière & C. Rivière		✗							
Poaceae	*Phyllostachys bambusoides*	Siebol & Zucc.		✗							
Poaceae	*Phyllostachys flexuosa*	A. & C. Rividre		✗							
Poaceae	*Poa annua*	L.					✗				
Poaceae	*Rottboellia cochinchinensis*	(Lour.) Clayton		✗	✗	✗					✗
Poaceae	*Setaria barbata*	(Lam.) Kunth					✗				
Poaceae	*Sorghum halepense*	(L.) Pers.		✗	✗		✗	✗			
Poaceae	*Sporobolus tenuissimus*	(Schrank) Kuntze		✗							
Poaceae	*Themeda arguens*	(L.) Hack.				✗					
Poaceae	*Themeda quadrivalvis*	(L.) Kuntze			✗						
Poaceae	*Trachypogon spicatus*	(L.f) Kuntze		✗							
Poaceae	*Tragus berteronianus*	Schult.					✗	✗			
Poaceae	*Tripsacum dactyloides*	(L.) L.		✗							
Poaceae	*Tripsacum latifolium*	Hitch.		✗							
Poaceae	*Urochloa arrecta*	(Hack. ex T. Durand & Schinz) Morrone & Zuloaga					✗				

Poaceae	*Urochloa distachya*	(L.) T.Q. Nguyen		✗							
Poaceae	*Urochloa mutica*	(Forssk.) T.Q. Nguyen		✗	✗		✗	✗		✗	✗
Poaceae	*Urochloa panicoides*	P. Beauv.		✗							
Poaceae	*Urochloa reptans*	(L.) Stapf					✗	✗			
Poaceae	*Zoysia matrella*	(L.) Merr.		✗							
Polygonaceae	*Antigonon leptopus*	Hook. & Arn.	✗	✗	✗		✗	✗		✗	
Polygonaceae	*Persicaria chinensis*	(L.) H.Gross				✗					
Polygonaceae	*Rumex acetosella*	L.			✗						
Polygonaceae	*Triplaris americana*	L.		✗							
Pontederiaceae	*Eichhornia azurea*	(Sw.) Kunth		✗	✗						
Pontederiaceae	*Eichhornia crassipes*	(Mart.) Solms	✗	✗	✗	✗	✗	✗	✗		
Pontederiaceae	*Eichhornia paniculata*	(Spreng.) Solm		✗							
Portulacaceae	*Portulaca oleracea*	L.		✗							
Potamogetonaceae	*Potamogeton diversifolius*	Raf.		✗							
Primulaceae	*Ardisia elliptica*	Thunb.					✗		✗		
Primulaceae	*Ardisia humilis*	Vahl		✗							
Primulaceae	*Ardisia solanaceae*	(Poir.) Roxb.				✗					
Protaceae	*Grevillea robusta*	A. Cunn. ex R.BR.				✗					
Rhamnaceae	*Colubrina asiatica*	(L.) Brongn	✗								
Rhamnaceae	*Ziziphus mauritiana*	Lam.				✗				✗	
Rosaceae	*Eriobotrya japonica*	(Thunb.) Lindl.									✗
Rosaceae	*Pyracantha coccinea*	M. Roem.		✗							
Rosaceae	*Rubus ellipticus*	Sm.				✗					
Rosaceae	*Rubus rosifolius*	Sm.		✗			✗		✗		
Rosaceae	*Rubus niveus*	Thunb		✗	✗						
Rosaceae	*Spiraea chamaedryfolia*	L.			✗						
Rubiaceae	*Ixora thwaitesii*	(Thwaites) Hook.f.		✗							
Rubiaceae	*Morinda citrifolia*	L.		✗	✗						

Rubiaceae	*Oldenlandia corymbosa*	L.					✗	✗			
Rubiaceae	*Oldenlandia herbacea*	(L.) Roxb.									✗
Rutaceae	*Glycosmis parviflora*	(Sims) Little		✗							
Rutaceae	*Triphasia trifolia*	(Burm. f.) P. Wilson					✗	✗	✗		
Salicaceae	*Flacourtia indica*	(Burm. f.) Merr.									✗
Sapindaceae	*Cardiospermum halicacabum*	L.		✗							
Sapindaceae	*Melicoccus bijugatus*	Jacq.					✗	✗			
Sapotaceae	*Manilkara zapota*	(L.) P. Royen									✗
Scrophulariaceae	*Bontia daphnoides*	L.		✗							
Scrophulariaceae	*Verbascum thapsus*	L.			✗						
Solanaceae	*Brugmansia candida*	Pers.		✗							
Solanaceae	*Brugmansia suaveolens*	(Humb. & Bonpl. ex Willd.) Bercht. & J.Presl			✗						
Solanaceae	*Cestrum diurnum*	L.	✗								
Solanaceae	*Datura stramonium*	L.		✗							
Solanaceae	*Nicotiana glauca*	Graham		✗	✗						
Solanaceae	*Nicotiana plumbaginifolia*	Viv.		✗							
Solanaceae	*Solanum elaeagnifolium*	Cav.		✗			✗	✗			
Solanaceae	*Solanum erianthum*	D. Don		✗							
Solanaceae	*Solanum mammosum*	Dunal		✗							
Solanaceae	*Solanum quitoense*	Lam.			✗						
Solanaceae	*Solanum seaforthianum*	Andrews		✗			✗	✗			
Solanaceae	*Solanum viarum*	Dunal					✗				
Sphenocleaceae	*Sphenoclea zeylanica*	Gaertn.		✗	✗		✗				
Tiliaceae	*Corchorus hirtus*	L.		✗							
Tiliaceae	*Triumfetta bogotensis*	DC.		✗							
Tiliaceae	*Triumfetta rhomboidea*	Jacq.		✗							
Urticaceae	*Urtica urens*	L.		✗							
Verbenaceae	*Citharexylum ellipticum*	Sessé & Moc.		✗							

Verbenaceae	*Lantana camara*	L.	✗		✗						✗
Verbenaceae	*Verbena rigida*	Spreng.		✗							
Vitaceae	*Ampelopsis arborea*	(L.) Rusby		✗							
Vitaceae	*Parthenocissus quinquefolia*	(L.) Planch.		✗							
Xanthorrhoeaceae	*Aloe vera*	(L.) Burm. f.		✗			✗	✗		✗	
Zingiberaceae	*Alpinia purpurata*	(Vieill.) K. Schum.		✗			✗	✗			
Zingiberaceae	*Alpinia zerumbet*	(Pers.) B.L. Burtt & R.M. Sm.		✗		✗	✗	✗			
Zingiberaceae	*Costus speciosus*	(J. Koning) Sm.		✗							
Zingiberaceae	*Costus spicatus*	(Jacq.) Sw.		✗							
Zingiberaceae	*Curcuma zanthorrhiza*	Roxb.							✗		
Zingiberaceae	*Etlingera elatior*	(Jack) R.M. Sm.					✗				
Zingiberaceae	*Hedychium coccineum*	Buch. Ham. ex Sm.				✗					
Zingiberaceae	*Hedychium coronarium*	J. König		✗		✗	✗		✗		
Zingiberaceae	*Hedychium gardneranum*	Sheppard ex Ker Gawl.				✗					
Zingiberaceae	*Zingiber capitatum*	Roxb.		✗							
Zingiberaceae	*Zingiber montanum*	(J. König) Link ex A. Dietr.					✗				
Zingiberaceae	*Zingiber officinale*	Roscoe		✗							
Zingiberaceae	*Zingiber zerumbet*	(L.) J.E. Sm.					✗				
Zygophyllaceae	*Tribulus cistoides*	L.					✗	✗			
